# Determinants of Chain Selection and Staggering in Heterotrimeric Collagens: A Comprehensive Review of the Structural Data

**DOI:** 10.3390/ijms262010134

**Published:** 2025-10-18

**Authors:** Luigi Vitagliano, Nunzianna Doti, Nicole Balasco

**Affiliations:** 1 Institute of Biostructures and Bioimaging, National Research Council (CNR), 80131 Naples, Italy; nunzianna.doti@cnr.it; 2 Institute of Molecular Biology and Pathology, CNR c/o Department of Chemistry, Sapienza University of Rome, 00185 Rome, Italy

**Keywords:** collagen heterotrimers, non-collagenous regions, chain recognition, structural biology, AlphaFold, protein folding, sequence–structure–function relationships, collagen staggering

## Abstract

Collagen is a family of large, fibrous biomacromolecules common in animals, distinguished by unique molecular, structural, and functional properties. Despite the relatively low complexity of their sequences and the repetitive conformation of the triple helix, which is the defining feature of this family, unraveling sequence–stability and structure–function relationships in this group of proteins remains a challenging task. Considering the importance of the structural aspects in collagen chain recognition and selection, we reviewed our current knowledge of the heterotrimeric structures of non-collagenous (NC) regions that lack the triple helix sequence motif, Gly-X-Y, and are crucial for the correct folding of the functional states of these proteins. This study was conducted by simultaneously surveying the current literature, mining the structural database, and making predictions of the three-dimensional structure of these domains using highly reliable approaches based on machine learning techniques, such as AlphaFold. The combination of experimental structural data and predictive analyses offers some interesting clues about the structural features of heterotrimers formed by collagen NC regions. Structural studies carried out in the last decade show that for fibrillar collagens (types I, V, XI, and mixed V/XI), key factors include the formation of specific disulfide bridges and electrostatic interaction patterns. In the subgroup of collagens whose heterotrimers create supramolecular networks (types IV and VIII), available structural information provides a solid ground for the definition of the basis of the molecular and supramolecular organization. Very recent AlphaFold predictions and structural analyses of type VI collagen offer strong evidence of the specific domains in the NC region of the protein that are involved in chain selection and their staggering. Insightful crystallographic studies have also revealed some fundamental elements of the chain selection process in type IX collagen. Collectively, the data reported here indicate that, although some aspects (particularly the quantification of the relative contribution of the NC and triple helix regions to correct collagen folding) are yet to be fully understood, the available structural information provides a solid foundation for future studies aimed at precisely defining sequence–structure–function relationships in collagens.

## 1. Background

The vast diversity of the protein universe is often analyzed and characterized using various molecular and structural features [1]. Among other classifications, those based on dynamic and structural properties are commonly applied. As a result of their conformational properties, proteins are widely categorized into three main types: fibrous, globular, and intrinsically disordered [2,3,4]. This scale of decreasing flexibility is reflected in precise sequence patterns. Indeed, only globular proteins fully utilize the chemical diversity of the twenty genetically encoded amino acids. In contrast, the sequences of fibrous proteins often display recurrent motifs, and those of intrinsically disordered proteins exhibit low-complexity compositions with an abundance of charged residues and a scarcity of hydrophobic residues.

Collagens are biomacromolecules that are generally classified as fibrous proteins and are highly abundant in animals, characterized by unique molecular, structural, and functional properties [5,6,7,8,9]. Collagens are intricate trimeric proteins formed through the assembly of large polypeptide chains, often comprising more than 1000 residues. The collagens’ distinctive feature is the triple helix structure whose formation relies on a precise repetitive sequence pattern: Gly-X-Y. Although positions X and Y can accommodate all amino acids, they are primarily occupied by imino acids, such as proline and hydroxyproline. Nevertheless, the collagens’ folding and functioning strongly depend on the presence in its sequence of unstructured regions and globular domains present in their sequences. Therefore, a complete understanding of collagen sequence-structure–function relationships requires an accurate definition of the interplay between these structurally distinct regions.

The structural characterization of collagen triple helix domains (THDs) has been a focus of extensive research over the past thirty years [5,6,7,8,9,10,11,12,13,14,15,16,17,18,19]. Despite the relatively simple sequences of THDs and the repetitive nature of their structural motifs, understanding the relationship between sequence, stability, and structure–function has proven to be a complex task [20]. An illuminating example is the elucidation of the structural basis by which the replacement of proline with the closely related hydroxyproline in the Y position confers stability to collagen, which is essential for proper functioning, requiring remarkable efforts [5,6,7,21,22]. The complexity of collagen is further increased by the staggering of the three chains within the triple helix, which positions the small-sized Gly at the center of the helix. Even when the triple helix comprises three identical polypeptide chains, they can be structurally arranged in three distinct configurations. Typically, the three staggered chains are known as leading (staggered toward the N-terminus), middle, or trailing (staggered toward the C-terminus). Additionally, things become more complicated when non-identical chains form the triple helix. For assemblies made up of two chains with a 2:1 stoichiometry, the number of combinations increases. For 1:1:1 heterotrimers, the number of possible combinations can reach as high as 27.

Although the quantification of their role is still debated, non-collagenous domains, i.e., the regions of collagen sequences lacking the recurrent Gly-X-Y sequence, play a crucial role in the selection and in the staggering of chains in functional collagens [23,24,25]. We here reviewed our current structural knowledge of NC regions in collagen and how they govern the correct triple helix assembly in collagens made up of different chains (heterotrimeric collagens) [26,27]. This study was conducted using an innovative approach that combines information retrieved from literature with data mined from structural databases, which is then integrated with recent and effective predictive methods, such as AlphaFold [28,29].

In the following paragraphs, we initially summarize the molecular features of collagen subclassing by focusing on heterotrimeric collagens. Then, some insights into the rules that play a role in collagen hetero-trimerization were derived by a global comparison of the sequences involved in this process. We then surveyed the structural information of collagen NC domains by integrating literature and database information with predictive analyses. This was arranged *per* collagen subfamily and then, *per* collagen type.

## 2. The Molecular and Functional Diversity of the Collagen Superfamily

Collagens are the primary protein components of the metazoan extracellular matrix [30]. Interestingly, despite several decades of active research on these biomacromolecules, strict classification criteria for identifying a protein as a collagen have yet to be defined [31,32,33,34,35]. Indeed, there are two distinct approaches to defining collagen(s). Although it is generally accepted that the defining feature of collagen is the triple helix and the associated Gly-X-Y motif underlying it, most studies restrict the term collagen to proteins that play a structural role in the extracellular matrix [33,34]. Alternatively, the term collagen is extended to include all proteins containing the triple helix motif, thereby encompassing intracellular proteins involved in the immune system or transmembrane signaling.

Even within the most restrictive definition adopted here, members of the collagen family exhibit significant variability in length, molecular weight, chemical composition, and how they interact to form supramolecular assemblies [36]. Recent reports indicate that humans possess collagen genes encoding 46 polypeptide chains, known as α-chains, which self-assemble into 28 distinct collagen types (I-XXVIII) [35,37]. Depending on their function, structure, and localization, collagens are grouped into different clusters, including those that form fibers, anchoring fibrils, beaded filaments, supramolecular networks, multiplexins, FACITs (fibril-associated collagens with interrupted triple helices), and MACITs (membrane-associated collagens with interrupted triple helices). Although most of these proteins operate as homotrimers, some of the most widespread collagens (types I, IV, V, VI, VIII, IX, and XI) form heterotrimers in their functional states. Notably, heterotrimer formation has been observed in collagens with vastly different supramolecular and functional properties. For instance, types I, V, and XI are fibrillar collagens [38], while types IV and VIII belong to collagens that create supramolecular networks. Types VI and XI are involved in forming beaded filaments and FACITs, respectively. The mechanisms by which these proteins favor hetero-interaction over self-interaction are a puzzling issue that is being thoroughly investigated. Several studies in the literature have shown that NC domains play a significant role in chain selection, leading to the formation of heterotrimers and their organization in functional collagen [23,37,39]. Recent studies, however, highlight that the correct assembly of collagen also depends on the combined action of NC and THD regions [40,41]. In fibrillar collagens, the precursor (procollagen) contains a C-terminal large non-triple helical propeptide domains that serve an analogous role in the chain selection and staggering as the NC domains of the other collagens. Considering the importance of the structural aspects in collagen chain recognition, we reviewed our current knowledge of the heterotrimeric structures of NC heterotrimers.

## 3. Heterotrimeric Procollagens: Analysis of the Sequence Similarities

As mentioned earlier, NC regions are essential for the correct assembly of collagen homo- and heterotrimers. Since this survey specifically examines collagen heterotrimerization, we analyzed and discussed the overall sequence similarities of the NC domains among collagen types that form heterotrimers (I, IV, V, VI, VIII, IX, and XI). In the next section, the analysis is organized by grouping these collagens based on their functional properties. Specifically, they are categorized into fibrillar (types I, V, and XI), network-forming (types IV and VIII), beaded filament-forming (type VI), and FACITs (type IX).

### Pairwise Sequence Comparisons

As reported in Table 1, collagen heterotrimers often display multiple stoichiometries. Although identifying the chains involved in collagen formation is an ongoing process, especially for rare collagens, there is significant variability in the stoichiometry of heterotrimers formed by different collagen types. Indeed, a single heterotrimeric species is formed for types I and IX. In contrast, collagens IV and VI exhibit three distinct heterotrimers. Occasionally, heterotrimers may result from mixing chains of different types (V and XI).

Since the overall sequence similarities of the NC regions may be an important factor in the observed associations, the following paragraph analyzes the percentage of sequence identity between pairs of NC regions in the chains that form the same heterotrimer. The definition of these regions for each collagen type is listed in Table S1. At the same time, the number of residues that were correctly aligned in the pairwise comparisons is reported in either the text or Tables S2–S5.

Among fibrillar collagens, in type I, the two chains (α1(I) and α2(I)) exhibit a sequence identity of 63.2%, based on 234 shared NC1 residues. Type V collagen forms both homotrimers [α1(V)]_3_ and heterotrimers [α1(V)]_2_α2(V) and α1(V)α2(V)α3(V). The chains involved in both the 1:2 and the 1:1:1 heterotrimers exhibit relatively low sequence identities, ranging from 35.6% to 53.9% (Table S2). The lowest sequence identity is observed between the pairs α2(V) and α3(V), which are components of the 1:1:1 heterotrimer. Type XI collagen forms the 1:1:1 heterotrimer α1(XI)α2(XI)α3(XI) [42]. The α3(XI) chain shares the same sequence as the α1 chain of type II collagen; however, it differs in its post-translational modifications and cross-linking patterns. In the α1(XI)α2(XI)α3(XI) heterotrimer, the NC1 domains of the three chains exhibit pairwise sequence identities ranging from 38.3% to 45.8% (Table S2). In mature articular cartilage, type XI collagen comprises a significant fraction of α1(V) chains, indicating the presence of V/XI hybrid molecules [43,44], with an α1(XI)α1(V)α3(XI) composition. The NC1 of α1(V) shares a significant sequence similarity with the corresponding region of α1(XI) (identity 78.2% in 229 overlap) and α3(XI) (identity 44.3% in 235 overlap). Similarly, the NC1 of α1(XI) presents analogies with α2(V) (identity of 39.3% in 234 overlap) (Table S2). Not surprisingly, in some tissues or cell lines, the α1(XI)/α3(XI) NC1 domains are associated with the α1(V)/α2(V) forming the heterotrimers α1(XI)α1(V)α3(XI) and [α1(XI)]_2_α2(V). Based on these observations, it has been proposed that V and XI chains in fact constitute a single collagen type in which different combinations of chains can associate in a tissue-specific manner [45].

Among network-forming collagens, type IV can be assembled from six different chains, with overall sequence identities of the NC1 domains ranging from 54% to 83.6% (Table S3). Notably, the heterotrimers with a 2:1 stoichiometry ([α1(IV)]_2_α2(IV) and [α5(IV)]_2_α6(IV)) have quite similar chains, with identities of α1(IV)/α2(IV) and α5(IV)/α6(IV) being 63.8% and 64.0%, respectively. In contrast, for the 1:1:1 heterotrimer α3(IV)α4(IV)α5(IV), the chains can show identities as low as 54%. In type VIII, two chains, α1(VIII) and α2(VIII), co-assemble to form both the 1:2 and 2:1 heterotrimers. These two chains exhibit quite similar sequences (72.7% identity, based on 128 shared residues of the NC1).

In the remaining types of collagens that form heterotrimers (types VI and IX), only assemblies with a 1:1:1 stoichiometry are observed. As seen with other 1:1:1 heterotrimers, the chains involved in types VI and IX assemblies also show relatively low sequence identities. Specifically, in the heterotrimer α1(IX)α2(IX)α3(IX), the sequence identities among the NC2 domains of the three chains range from 29.6% to 53.8% (Table S4). Lower sequence similarities are observed in the heterotrimers α3(VI)α2(VI)α1(VI) (range 20.1–31.7%) and α5(VI)α2(VI)α1(VI) (range 16.3–31.7%) when the first two C-terminal vWF-A-like subdomains (C1 and C2) are compared (Table S5). Greater sequence differences are seen in the α2(VI) and α6(VI) chains that participate in the trimer α6(VI)α2(VI)α1(VI), as no acceptable alignment can be obtained for this region.

This analysis shows that as heterotrimers become more molecularly complex, changing from a 2:1 to a 1:1:1 stoichiometry, their sequence similarities decrease. This suggests that the selection of three different chains necessitates specific interactions, which are facilitated through significant sequence changes.

The data reported here indicate that collagens achieve hetero-trimerization through chains that generally show remarkable similarities. In fact, it has been noted that the average sequence identity between interacting homologous subunits is only 23–24%, a value that is often exceeded in collagen heterotrimers.

## 4. Heterotrimeric Procollagens: Structural Data

Because the understanding of chain recognition in collagen heterotrimers heavily relies on atomic-level structural data, we examined the Protein Data Bank (PDB, release of June 2025) [46] to find experimentally determined three-dimensional models of the NC regions of the specific collagen types studied here (Table 1). Since insights into the hetero-oligomerization process can also be derived from the structure of homotrimers, if available, these models were included in Table 1 as well. Since experimental structural information is missing for many of these NC domains, we filled this gap by performing predictions using the AlphaFold3 (AF3) algorithm (https://alphafoldserver.com/, accessed on 1 June 2025) with default settings [29]. The best predicted model (model 0) out of the five computed by AF3 was considered throughout the present work.

### 4.1. Survey of the PDB and AlphaFold Predictions

**Figure 1:**
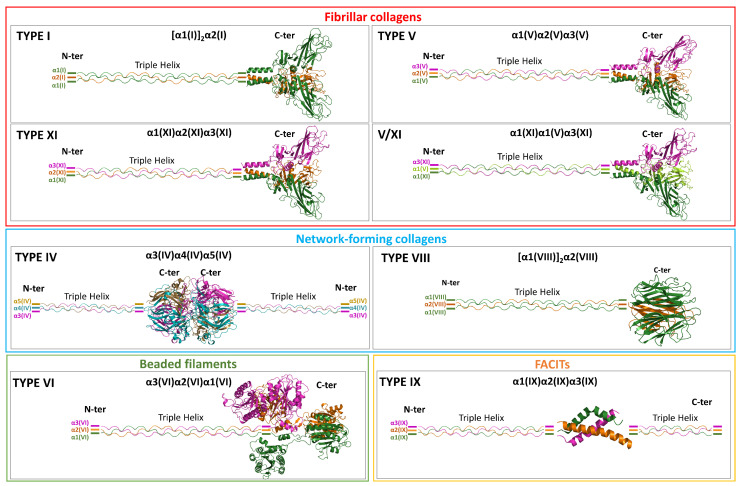
Schematic representation of the heterotrimeric assemblies formed by collagens within each subfamily. For collagen types that form more than one heterooligomeric association, the assembly displaying the highest variability is shown. When available, crystallographic models of the NC assemblies were used (PDB ID: 6wku for type IV and 5ctd for type IX); for the remaining types, AF3-predicted models were employed.

The inspection of the PDB shows that some NC regions of collagens have been thoroughly structurally characterized (Table 1). This is especially true for type IV, where both homotrimeric and heterotrimeric forms have been documented at the atomic level. Structural studies have also shown how type IV collagen heterotrimers assemble into larger supramolecular structures (Figure 1). Heterotrimers have also been structurally analyzed for type IX. Structural data exist for the homotrimers of types I and VIII. Insights into the structure of heterotrimers of types V and XI can be inferred from data on the homologous type I. Lastly, there is no available structural information for type VI collagen.

As mentioned above, we integrated these analyses using AF3 predictions, which were critically evaluated for reliability. Specifically, the accuracy of the AF3 models was assessed by analyzing the predicted Template Modelling (pTM), the interface predicted Template Modelling (ipTM) scores, the per-residue Local Distance Difference Test (pLDDT), and the Predicted Aligned Error (PAE) matrices reported for each predicted structure [28,29]. The effectiveness of this approach was also evaluated on a case-by-case basis, considering its ability to reproduce available structural data or provide a structural framework for interpreting findings reported in the literature.

In the following paragraphs, available structural data are reported by grouping these collagens according to their global properties (fibrillar, network-forming, beaded filaments forming, and FACITs).

### 4.2. Heterotrimeric Fibrillar Collagens

#### 4.2.1. Type I

Type I collagen is part of the subclass of fibril-forming collagens [36,38,47,48]. This type is the most abundant collagen found in mammals and is widely used in basic studies to identify the general properties of collagen fibers, as well as in the development of collagen-based biomaterials for tissue engineering. It is primarily found as a heterotrimer composed of two α1(I) chains and one α2(I) chain, [α1(I)]_2_α2(I). The homotrimeric form of this protein, consisting of three α1(I) chains, occurs in embryonic tissues and more rarely in adult skin. For type I collagen, Hulmes and coworkers have determined the crystal structure of homotrimers of the procollagen C-propeptides of chain α1 (PDB ID: 5k31) [27]. Although this is not the most relevant biological oligomer, this arrangement has provided essential insights into the structural determinants of hetero-oligomerization for this collagen type.

Initial information on the structural features of this domain was obtained through homology with the crystallographic structure of the homotrimer α1(III) C-propeptide [49]. This structure revealed a previously unknown protein fold for the three chains, which assembled to form a trimer resembling a flower. It also highlighted the critical structural roles of Ca^2+^ ions and inter-chain disulfide bonds, along with an intrinsic asymmetry of the trimer believed to be vital for directing the staggering of the chains within the triple helix. The determination of the structure of the homotrimer formed by the α1(I) C-propeptide chains confirmed the role of Ca^2+^ and the disulfide bridges in stabilizing the trimeric structure [27]. Still, they did not observe the asymmetry that may be responsible for the staggering of the three chains (see also [50] for further details). By integrating these crystallographic data with molecular modeling, Hulmes and colleagues successfully identified key residues involved in the hetero-trimerization process. Specifically, they generated a tetramutant in which the formation of the [α1(I)]_2_α2(I) heterotrimer was abolished entirely.

**Figure 2:**
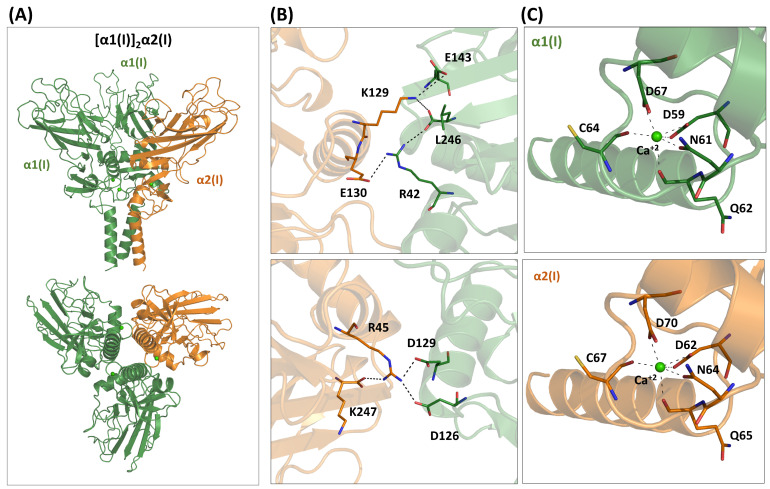
AF3-predicted model of the [α1(I)]_2_α2(I) heterotrimer formed by the NC1 domains of collagen type I chains. (**A**) Cartoon representation. (**B**) Stabilizing interactions at the hetero-interfaces. (**C**) Calcium ion coordination in chains α1(I) and α2(I).

To determine whether the newly available predictive approaches could replicate the experimental data and potentially provide further insights into the heterotrimerization process of these domains, we utilized AlphaFold to generate the structure of the [α1(I)]_2_α2(I) heterotrimer (Figure 2A). The predicted model provides scores for the AF3 self-evaluation assessment, particularly the ipTM (0.85) and the pLDDT values, as well as the PAE matrix, indicating a confident and high-quality prediction (Figure S1). It is important to note that AF3 cannot determine whether a chain can form a trimer. Indeed, for α2(I), which cannot form homotrimers, AF3 predicts a folded structure, although with a lower ipTM score (0.70). This observation is a consequence of the well-known bias of AF3 toward folded structures, even in the presence of local destabilizing interactions [51].

We evaluated the AF3 structure of the [α1(I)]_2_α2(I) to determine whether it recapitulates some of the experimentally confirmed information. The predicted model indicates that several polar and electrostatic interactions stabilize the heterotrimer (Figure 2B). This includes residues such as Arg45, Lys129, Glu130, and Lys247 of α2(I) (C-propeptide numbering, see Table S1). It is worth noting that similar considerations were derived by Sharma et al. [27] using standard molecular modeling techniques. The multiple interactions of Arg45 of the chain α2(I) observed in the AF3 model well fit with the observed destabilization of the heterotrimer upon its mutation to Ala [27].

The crystallographic structure of the [α1(I)]_3_ homotrimer has also emphasized the importance of calcium binding for stabilizing the protein [27]. AF3 accurately predicts the location and coordination of the Ca^2+^ metal in the α1(I) chain (Figure 2C and Figure S2). Since the residues of the coordination sphere are conserved in α2(I), the metal is also tightly bound to this chain of the heterotrimer (Figure 2C).

**Figure 3:**
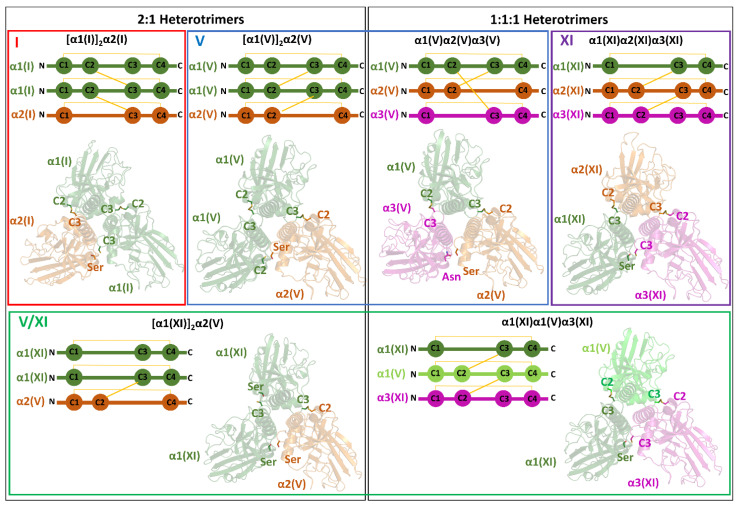
Schematic representation of the disulfide-bonding network in the NC1 domains of heterotrimeric fibrillar collagens. The AF3-predicted models of the different heterotrimers are shown as cartoons, with disulfide bridges depicted as sticks.

Previous studies have insightfully linked the ability of type I to form heterotrimers to the presence or absence of specific cysteine patterns in the sequence [39]. Specifically, a recurrent intermolecular disulfide bridge connects the second cysteine of the domain (C2) to the third (C3) of an adjacent chain. This pattern of three disulfide bridges stabilizes the homotrimers. Indeed, this is the configuration observed in the crystallographic structure of the [α1(I)]3 trimer [27]. Notably, according to Shoulders and colleagues [39], who corroborated an old hypothesis [52], the absence of C2 in the α2(I) in this pattern would favor the formation of [α1(I)]2α2(I) over the [α1(I)]3 homotrimer. The AF3 model accurately predicts the formation of the two disulfide bridges (α1C2-α1C3 and α1C2-α2C3) (Figure 3).

Collectively, these findings indicate that the stabilization of the functional [α1(I)]_2_α2(I) is due to the combination of the formation of specific disulfide bridges and electrostatic interactions at the α1(I)-α2(I) hetero-interfaces, therefore indicating that the disulfide bonding is just one factor in a complex process that involves other factors.

#### 4.2.2. Type V

Type V is a minor component of the extracellular matrix that has been described as a regulatory fibril-forming collagen [53,54]. It has been detected in both homomeric ([α1(V)]_3_) and heteromeric states ([α1(V)]_2_α2(V) and α1(V)α2(V)α3(V)). It has also been found in combination with type XI collagen (α1(XI)α1(V)α3(XI) and [α1(XI)]_2_α2(V)).

The inspection of the PDB shows that no structure has been reported for any chain of type V. However, it is worth noting that the NC1 domains of the three chains of this collagen are related to α1(I) and, to a lesser extent, α2(I). The sequence identities of α1(I) NC1 with the chains α1(V), α2(V), and α3(V) are 43.6% (236 overlap), 61.1% (234 overlap), and 38.7% (235 overlap), respectively. Therefore, the main structural features of the heterotrimers formed by type V ([α1(V)]_2_α2(V) and α1(V)α2(V)α3(V)) are expected to resemble those seen in the trimers formed by the NC1 of type I. In this scenario, we predicted the AF3 structures of [α1(V)]_2_α2(V) and α1(V)α2(V)α3(V) (Figure 1), which present excellent AF3 self-evaluation scores (Figure S3). The overall fold of these trimers closely resembles the experimental homotrimer of the α1 C-propeptide of collagen I [27]. Indeed, the root mean square deviation (RMSD) value, calculated on the C^α^ atoms, for [α1(V)]_2_α2(V) and α1(V)α2(V)α3(V) compared with the type I homotrimer is 2.16 Å (630 superimposed C^α^ atoms) and 2.34 Å (626 superimposed C^α^ atoms), respectively. Accordingly, as with type I collagen, [α1(V)]_2_α2(V) and α1(V)α2(V)α3(V) display well-defined calcium binding sites. As shown in Figure S4, the side chains involved in the calcium coordination are the same as those detected in type I collagen.

As anticipated by DiChiara et al. [39], the disulfide bridge patterns observed in the AF3 structures strictly adhere to the rules proposed by Shoulders and colleagues. The presence of both C2 and C3 in the chain is in line with the ability of α1(V) to form a homotrimer. The expected three disulfide bridges linking the C2 and C3 Cys are observed in the AF3 structure of α1(V) homotrimer. In the heterotrimer with a 2:1 stoichiometry [α1(V)]_2_α2(V), the lack of the C3 Cys, which is replaced by a serine, in the α2 chain allows the formation of only two disulfide bonds (α1C2-α1C3 and α1C2-α2C3) (Figure 3). In the 1:1:1 heterotrimer α1(V)α2(V)α3(V), the simultaneous lack of α2C3 and α3C2, which is replaced by an Asn, leads to a pivotal role of the α1 chain, whose C2 and C3 make bonds with α2C3 and α3C2, respectively.

Interestingly, some of the residues, such as Arg45, Lys129, Glu130, and Lys247 of chain α2 of collagen type I that play an important role in the stabilization of the heterotrimer, are not generally conserved in the sequences of the chains involved in the type V heterotrimers. This suggests that different electrostatic/polar interactions stabilize type I and type V heterotrimers.

The inspection of AF3 models indicates that polar/electrostatic interactions nevertheless stabilize the heterotrimer interfaces. The heterotrimer [α1(V)]_2_α2(V) is stabilized by the interactions established by the side chain of the positively charged residue in position 28 (Lys in α1 and Arg in α2) with polar groups present in the side chain of the residue located in position 23 (Glu in α1 and Gln in α2). Notably, this heterotrimer is additionally stabilized by a salt bridge formed by Arg29 of α1(V) and Glu66 of α2(V), which cannot be formed in the homotrimeric associations, being the residue in position 29 of α2(V) a Ser and the residue in position 66 of α1(V) an Arg. The side chain of Arg66 in α1(V) forms hydrogen bonding interactions with the main-chain oxygen of Leu 46 and Cys 47 at the other two heterotrimer interfaces. All three interfaces are stabilized by an additional interaction formed by the side chain of the conserved Asp43 with the main-chain nitrogen of the residue in position 64 (Cys in α1 and Ser in α2)

In the heterotrimer α1(V)α2(V)α3(V), a similar pattern of interactions is observed due to the similarity of the α3(V) and the α1(V) chains. In addition to the global identity (54%, see Table S2), the two chains share most of the residues that form crucial interactions at the interface, such as Glu23, Arg28, Arg29, and Glu43. In this framework, the α1(V)α2(V)α3(V) assembly may be considered as a surrogate of [α1(V)]_2_α2(V) with the simple replacement of one of the two α1(V) with α3(V). The heterotrimer is, however, stabilized by an additional electrostatic interaction formed by the side chain of Asp67 of α2(V) with Arg46 of α3(V). This interaction can be exclusively formed at the α2/α3 interface since in the α1 and α2 chains, the residue in position 46 is a Leu.

Collectively, these data highlight the interplay in stabilizing these trimers between disulfide bridges and polar/electrostatic interactions.

#### 4.2.3. Type XI

Type XI collagen is a minor fibril component in tissues where type II collagen is predominant. It is observed in both normal and disease conditions. In cartilaginous tissues, collagen XI exists as heterotrimers with an α1(XI)α2(XI)α3(XI) stoichiometry [55]. Notably, the pro-α3(XI) chain is the same gene product as the pro-α1(II) chain, and it assembles with the other chains after extensive post-transcriptional modifications. The inspection of the PDB shows that no structural characterization has been conducted on type XI collagen. The AF3 prediction for the heterotrimer α1(XI)α2(XI)α3(XI) yields good validation scores and exhibits the expected structural similarity to the experimental structure of the homotrimer of pro-α1(I) (RMSD value of 1.59 Å, 572 superimposed C^α^ atoms) (Figure S5). As for the other fibrillar collagens here analyzed, AF3 suggests the presence of a calcium ion per chain, which displays a conserved coordination sphere (Figure S4).

The sequences of the NC1 of α2(XI) and α3(XI) chains present both the C2 and C3 Cys, whereas α1(XI) lacks C2, which is replaced by a Ser. In the AF3 structure of this heterotrimer, a pivotal role is played by α2 chain, whose C2 and C3 residues form bonds with α1C3 and α3C2, respectively (Figure 3). Although heterotrimers with 2:1 stoichiometry are in principle possible according to the disulfide hypothesis, they have not been observed.

The inspection of the interchain interfaces of the AF3 model for α1(XI)α2(XI)α3(XI) indicates that limited polar interactions occur. The only strong contacts are the electrostatic interactions formed by the side chains of Asp23(α1)-Arg28(α2), Glu23(α2)-Arg28(α3), and Glu141(α2)-Arg186(α3).

#### 4.2.4. Mixed Type V/XI

The characterization of isolated from mature articular cartilage indicates that type XI collagen includes a significant fraction of α1(V) chains [44], implying the presence of V/XI hybrid molecules. Indeed, with maturation of articular cartilage, the α1(V) chain progressively replaces the α2(XI) chain. The formation of mixed molecular isoforms characterized over the years indicates the presence of two mixed hybrid heterotrimers, α1(XI)α1(V)α3(XI) and [α1(XI)]_2_α2(V) [43,45]. In the mixed NC1 heterotrimer α1(XI)α1(V)α3(XI), both the α3(XI) and α1(V) chains contain the C2 and C3 cysteines. In contrast, as mentioned above, α1(XI) lacks C2. Regarding disulfide bridges, the AF3 model shows similarities with the heterotrimer α1(XI)α2(XI)α3(XI) (Figure S6). In this structure, both the α2(XI) and α3(XI) chains contain C2 and C3 cysteines, while α1(XI) again lacks C2, which is replaced by a serine. In α1(XI)α1(V)α3(XI) structure, the α1(V) chain plays a key role, as its C2 and C3 residues form bonds with α1(XI)C3 and α3(XI)C2, respectively (Figure 3). In the reported alternative hybrid association [α1(XI)]_2_α2(V) [45], it is not possible to link all three chains simultaneously with disulfide bridges because the two α1(XI) lack C2, while α2(V) lacks C3. In this case, a single disulfide bridge is formed between α1(XI)C3 and α2(V)C2 (Figure 3).

In terms of interactions, compared to the α1(XI)α2(XI)α3(XI), the mixed heterotrimers α1(XI)α1(V)α3(XI) and [α1(XI)]_2_α2(V) present a slightly larger number of interface electrostatic/polar contacts. In addition to the interactions observed between residues in positions 23 and 28, in these heterotrimers, the side chain of the conserved residue Asp43 links the main chain nitrogen of the residue in position 64. In addition, the structure of the α1(XI)α1(V)α3(XI) is also stabilized by the salt bridge formed by Asp67(α1(V))-Arg42(α3(XI)).

### 4.3. Heterotrimeric Network-Forming Collagens (Types IV and VIII)

In the widely diverse modes in which collagen manifests, types IV, VIII, and X are classified in the network-forming group as they form open networks rather than fibers [37,56]. These collagens provide molecular scaffolds and interact with cells, growth factors, and other basement membrane components such as laminin, nidogen, and perlecan [57]. Here, the attention is focused on the structural organization of the NC1 domains of types IV and VIII, which associate as heterotrimers.

#### 4.3.1. Type IV

Type IV collagen belongs to the network-forming collagen subfamily and is present in basement membranes [45,57,58]. To date, six α-polypeptide chains, α1(IV)-α6(IV), have been identified. Each type IV chain features a short N-terminal collagenous 7S domain, a central collagenous region of approximately 1300–1400 residues, and a C-terminal globular non-collagenous (NC1) domain of roughly 225 residues (Table S1).

Despite the large number of possible heterotrimeric states, in which six distinct chains could be involved, only three combinations have been found in nature, i.e., the heterotrimers [α1(IV)]_2_α2(IV), α3(IV)α4(IV)α5(IV), and [α5(IV)]_2_α6(IV) [58]. This observation suggests that an important chain selection process, yet to be fully clarified, operates in the type IV collagen folding.

Type IV NC1 regions have been extensively studied from a structural perspective. The crystal structure has been reported for several homo- and hetero-oligomers (Table 1) [59,60,61,62,63]. These studies have provided an atomic-level description of these assemblies and of the mechanism of their trimerization and their subsequent association in large supramolecular networks. These results have been comprehensively illustrated in recent reviews [37,64]. Therefore, we only briefly summarize the main findings reported in the literature, referring readers to these reviews for further details.

The inspection of the literature and of the PDB indicates that the first structural characterization of this NC1 was conducted on the human and bovine [α1(IV)]_2_α2(IV) heterotrimers (Table 1) [59,60,61]. These structural characterizations highlighted the propensity of these domains to form hexameric structures by the juxtaposition of the two heterotrimer domains. Over the years, several studies provided strong evidence of the key role that negative ions and, in particular, chloride, play in the hexamerization process. The role of this anion was initially assessed by using a plethora of biochemical/biophysical techniques [64,65]. More recently, crystal structures of Cl^−^ bound NC1 homo- and hetero-hexamers [α1(IV)]_2_α2(IV), [α1(IV)]_3_, [α3(IV)]_3_, and [α5(IV)]_3_ have revealed the number and positions of Cl^−^ ions within each canonical hexamer [62,63]. The localization of the anions was favored by the use of elevated concentrations of Cl^−^ in the protein purification and crystallization procedure [63]. These twelve anions were classified into groups 1 and 2 according to their localization and coordination. Interestingly, it has been found that aliphatic C-H groups contribute to the coordination of group 2 chloride, which presents a highly dynamic behavior [64]. In contrast, the crystal structures of [α2(IV)]_3_ and [α4(IV)]_3_ showed that the NC domains of these chains naturally form homo-oligomers that differ from the typical hexameric structure, resulting in higher-order assemblies like dimers of tetramers and dimers of hexamers, respectively [62].

**Figure 4:**
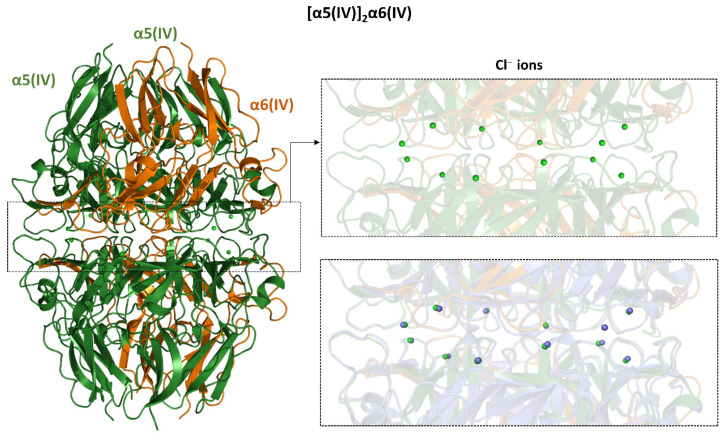
Cartoon representation of the AF3-predicted model of the [α5(IV)]_2_α6(IV) heterotrimer formed by the NC1 domains of collagen type IV. The top-right panel shows a zoomed view of the interface between the two trimers within the hexamer, highlighting chloride ions depicted as green spheres. The bottom panel displays the same region superimposed with the crystallographic structure of the α3(IV)α4(IV)α5(IV) heterooligomer (violet, PDB ID: 6wku).

Considering the role that chloride ions have in the correct assembly of the NC1 domain of type IV collagen, we evaluated the ability of AF3 to predict their location in the structure of the NC1 hexamers of three naturally detected hetero-oligomers. As shown in Figures S7 and S8, AF3 was able to correctly identify the position of all twelve anions present in the structure of [α1(IV)]_2_α2(IV) (PDB ID: 6mpx) [63] and α3(IV)α4(IV)α5(IV) (PDB ID: 6wku) [66], including those belonging to group 2, which are endowed with a dynamic behavior. Although not surprisingly, considering the remarkable sequence identities among type IV chains, AF3 was also able to identify the location of the twelve chlorides that decorate the structure of [α5(IV)]_2_α6(IV) (Figure 4), whose crystallographic structure is not currently available.

#### 4.3.2. Type VIII

Type VIII is a nonfibrillar collagen whose supramolecular assembly results in the formation of a hexagonal lattice, a feature that is shared with type X collagen [67]. Functional type VIII collagen is created by the combination of two independent chains, α1(VIII) and α2(VIII), which, as mentioned earlier, show remarkable sequence similarities (72.7% identity). These two chains may form homotrimers or heterotrimers. The heterotrimers present a 2:1 stoichiometry ([α1(VIII)]_2_α2(VIII) and α1(VIII)[α2(VIII)]_2_) [68]. In both chains, the central triple helix domain is surrounded by two non-collagenous regions at the N- (NC2) and C-terminus (NC1), with the latter belonging to the C1q-like family and forming a stable trimer.

**Figure 5:**
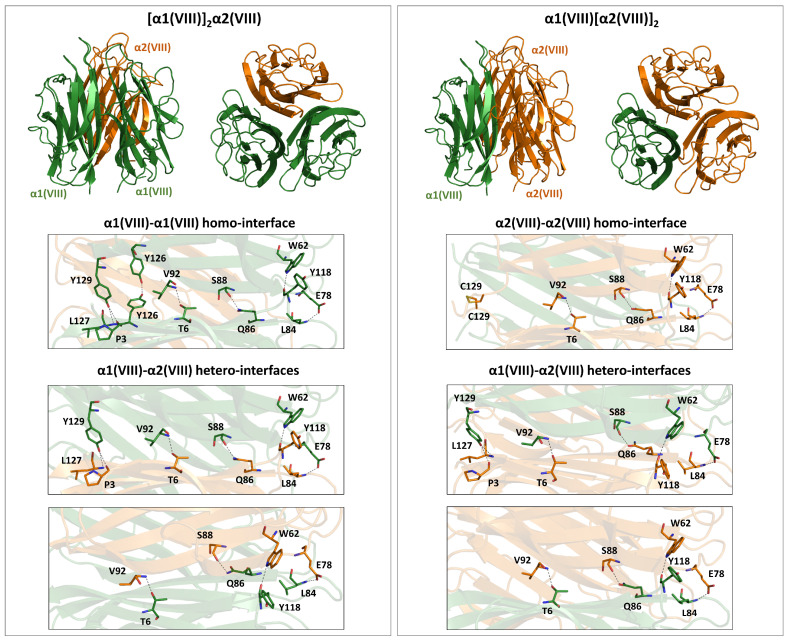
AF3-predicted models of the [α1(VIII)]_2_α2(VIII) and α1(VIII)[α2(VIII)]_2_ heterotrimers formed by the NC1 domains of collagen type VIII chains. Key stabilizing hydrogen-bond interactions at both the homo- and hetero-interfaces are shown.

The inspection of the PDB shows that only the mouse [α1(VIII)]_3_ NC1 domain trimer has been structurally characterized (PDB ID: 1o91) (Table 1) [69]. In this structure, each subunit folds into a ten-stranded beta-sandwich. The three interfaces of the homotrimer are stabilized by a network of polar interactions, which include hydrogen-bonding interactions formed by either main- or side-chain atoms (Figure S9). As stated by the authors [69], although non-conservative amino acid substitutions between the α1(VIII) and α2(VIII) chains occur at the subunit interfaces, it is not clear from the structure how much they influence the preferred assembly of collagen VIII α1 and α2 chains into heterotrimers. To gain some insights into some structural determinants of the chain selection in type VIII, we predicted the structures of the two heterotrimers using AF3 (Figure S10). The inspection of the three-dimensional models of these two complexes shows that some interactions of the mouse homotrimer are retained at all interfaces (Figure 5 and Figure S9). However, the replacement of α1Tyr126 (position 738 of the mouse sequence) with α2Phe126 abolishes the hydrogen bonding interaction between two adjacent Tyr126 of the homotrimer in the heterotrimeric interface of [α1(VIII)]_2_α2(VIII) and α1(VIII)[α2(VIII)]_2_. Similarly, the replacement of α1Tyr129 (position 741 of the mouse sequence) with α2Cys129 prevents the formation of the hydrogen bonds by the side chain of Tyr. However, the two α2Cys129 of the α1(VIII)[α2(VIII)]_2_ heterotrimer are spatially close to form a disulfide bridge (Figure 5). Therefore, at least in this latter heterotrimer, the loss of hydrogen bonding interactions of the homotrimeric association may be compensated by the disulfide bridge formation.

### 4.4. Heterotrimeric Beaded Filaments-Type VI

Type VI collagen is widely distributed throughout connective tissues [70,71]. In contrast to most other collagens, type VI collagen undergoes some polymerization before secretion. Indeed, heterotrimers formed by the association of three chains create dimers that then align their ends in register to form tetramers, which constitute the secreted form [72]. Tetramers then align end-to-end in the extracellular space to form type VI collagen microfibrils. Type VI collagen is composed of five different chains (α1, α2, α3, α5, and α6) that assemble to form the three heterotrimers (α3(VI)α2(VI)α1(VI), α5(VI)α2(VI)α1(VI), and α6(VI)α2(VI)α1(VI)) [73,74]. In all chains, the central triple helical domain is surrounded by globular domains at the N- and C-terminus. A global comparison of the molecular organization of these chains shows that α1(VI) and α2(VI) chains are significantly smaller and contain a single N-terminal subdomain (N1) and two globular domains at the C-terminus (C1 and C2). All of these domains are homologous to type A domains of von Willebrand factor (vWF-A). The α3(VI) chain has 10 N-terminal subdomains (N1-N10), two C-terminal vWF-A-like subdomains (C1 and C2), and three chain-specific C-terminal subdomains (C3-C5). The other two chains, α5(VI) and α6(VI), are more similar in size to α3(VI) than to α1(VI)/α2(VI), and they replace the former in the heterotrimeric assemblies. The α5(VI) and α6(VI) chains contain seven N-terminal domains (N1-N7). The α5(VI) chain contains two vWF-A-like subdomains (C1 and C2) and an additional domain. The α6 chains have five C-terminal domains, three of which are vWF-A-like.

**Figure 6:**
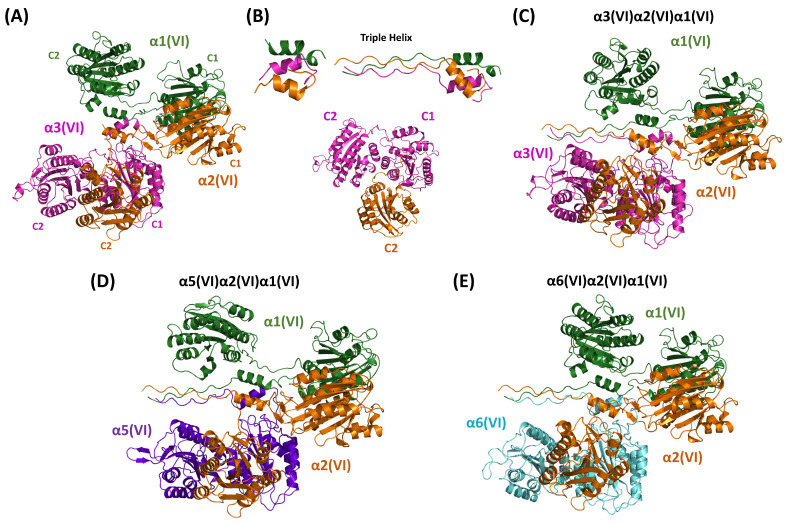
AF3-predicted models of the α3(VI)α2(VI)α1(VI) heterotrimer formed by (**A**) the C1-C2 domains of the three different chains and (**B**) the C2 domain of α2(VI) with C1-C2 of α3(VI) or the three-helix coiled-coil motif formed by the N-terminal regions of C1. AF3-predicted models of (**C**) α3(VI)α2(VI)α1(VI), (**D**) α5(VI)α2(VI)α1(VI), and (**E**) α6(VI)α2(VI)α1(VI), each formed by the C1-C2 domains of collagen VI chains including a THD portion at the N-terminus (See Table S1 for the residue renges).

In the survey of the PDB release of June 2025, no structural data were reported for the NC regions of collagen type VI. Therefore, we conducted AF3 predictions of the α3(VI)α2(VI)α1(VI) heterotrimer using the region including the two C-terminal vWF-A-like subdomains (C1-C2) of the different chains involved. The resulting models highlight the regions that interact with one another. Although the overall quality of the predicted interfaces of the complex, as assessed in terms of ipTM (0.5), falls into the gray zone, the model indicates significant interactions between specific regions of these chains (Figure 6A and Figure S11). Specifically, the strongest interactions are observed between C2 of α2(VI) and C1-C2 of α3(VI). Notably, when predictions are made using only these specific regions, an excellent ipTM value (0.88) is achieved, indicating high-quality predictions (Figure 6B and Figure S11). Moreover, the global complex also shows a three-helix coiled-coil motif formed by the N-terminal regions of these domains. This corresponds to residues 593–609, 591–607, and 2376–2392 for α1(VI), α2(VI), and α3(VI), respectively. Again, when the prediction is executed using only this region, an ipTM value of 0.6 is obtained (Figure 6B and Figure S11). Although this value falls on the borderline of corrected predictions, it is worth mentioning that hydrophobic interactions and potential disulfide bond bridges stabilize its trimeric interface. In the AF3 model, the cysteine residues of the CPCC motif of α3(VI) (residues 2387–2390) are spatially close to the CSCC motif of α1(VI) (residues 604–607) and the CGCC motif of α2(VI) (residues 602–605) (see below). This arrangement is confirmed when a triple helical region is added to this model (Figure 6B and Figure S11). The AF3 prediction suggests a staggering of the chain with the order α2α1α3.

We then assembled these fragments into a more comprehensive structure of these NC domains of the α3(VI)α2(VI)α1(VI) heterotrimer. As shown in Figure 6C, the structural features of the smaller assemblies are compatible with the formation of the larger assembly. As indicated by the PAE matrix (Figure S11), some reliable interactions are detected between the C1 domains of the chains α1(VI) and α2(VI). Among the structural features retained in this larger assembly, we detected the presence of disulfide bridges involving residues of the three-helix coiled-coil motif. In particular, the proximity of some residues of the Cyr-rich regions of these chains suggests the potential occurrences of the following disulfide bridges: α1(VI)Cys604-α3(VI)Cys2387, α2(VI)Cys602-α3(VI)Cys2390, α2(VI)Cys605-α3(VI)Cys2389, and α2(VI)Cys604-α3(VI)Cys2339.

These findings suggested to us to perform similar predictions on the other type VI heterotrimers in which the chain α3 is replaced by α5 (α5(VI)α2(VI)α1(VI), Figure 6D and Figure S11) or α6 (α6(VI)α2(VI)α1(VI), Figure 6E and Figure S11). Despite the limited sequence identity of chain α3 with chains α5 and α6 (approximately 33%-Table S5) and the absence of the CPCC motif displayed by α3(VI), the assemblies of the corresponding NC domains formed by α5(VI)α2(VI)α1(VI)) and α6(VI)α2(VI)α1(VI) closely resemble the one formed by α3(VI)α2(VI)α1(VI). The analogy of the PAE matrices suggests very similar interaction between the different domains. Interestingly, the absence of the CPCC motif in α5(VI) and α6(VI) does not prevent the formation of a network of disulfide bridges that stabilize the three-helix coiled-coil motif, which is contiguous to the triple helix structure. In α5(VI)α2(VI)α1(VI), the proximity of Cys residues in the AF3 model suggests the formation of the following bridges: α1(VI)Cys606-α2(VI)Cys605, α2(VI)Cys602-α5(VI)Cys1747, and α2(VI)Cys604-α5(VI)Cys1795. On the other hand, the α6(VI)α2(VI)α1(VI) is likely stabilized by the disulfide bonds α1(VI)Cys604-α2(VI)Cys605 and α2(VI)Cys604-α6(VI)Cys1794.

Collectively, these findings suggest that trimerization may start with the interactions between C2 of α2(VI) and C1 of α3(VI). This process is then propagated by weaker interactions among the other vWF-A domains up to the N-terminal fragments of the C-terminal domains, resulting in the assembly of a three-helix coiled-coil that properly positions the triple helix motif.

While we were finalizing the writing of this manuscript, the structures of heterotrimeric mini-collagen VI constructs were reported in the literature and deposited in the PDB (PDB IDs: 9gtu and 9han) [75]. The effective combination of AlphaFold prediction and cryo-EM data led to the definition of the medium-resolution structure of two assemblies of α3(VI)α2(VI)α1(VI). The smaller one comprises the coiled-coil motif of the three chains, the C2 domain of α2, and the C1/C2 domains of α3 (PDB ID: 9gtu) [75]. The larger assembly contains the dimeric association of the coiled-coil motif coupled with a triple helix region of the three chains, the C2 domain of α1 and α2, and the C1 domain of α3 (PDB ID: 9han) [75]. The data reported in the manuscript are generally in line with the data shown here for α3(VI)α2(VI)α1(VI), including the identification of the coiled-coil motif as a crucial factor for the heterotrimerization and its stabilization by disulfide bridges and the staggering of the three-chains. This determined microfibril structure provides a template for understanding supramolecular assembly of collagen VI [75].

### 4.5. Heterotrimeric FACITs-Type IX

Type IX is a member of the fibril-associated collagen subfamily (FACITs), which is present in the chondrocytes of growth-plate cartilage, adult articular cartilage, intervertebral discs, and the inner ear [76]. The Type IX collagen heterotrimer consists of α1(IX), α2(IX), and α3(IX) chains (α1(IX)α2(IX)α3(IX)), each containing three triple-helix domains that are interspersed and flanked by non-collagenous domains (NC1, NC2, NC3, and NC4). The inspection of the PDB reveals that the crystal structure of the type IX α1(IX)α2(IX)α3(IX) NC2 hetero-trimerization domain conjugated to some type I peptides, forming a triple helix motif, has been reported [77]. These remarkable structures have provided key insights into the chain selection and staggering processes occurring during heterotrimer formation [77]. In addition, the authors also report a robust method to produce fragments of hetero-trimeric collagenous regions.

**Figure 7:**
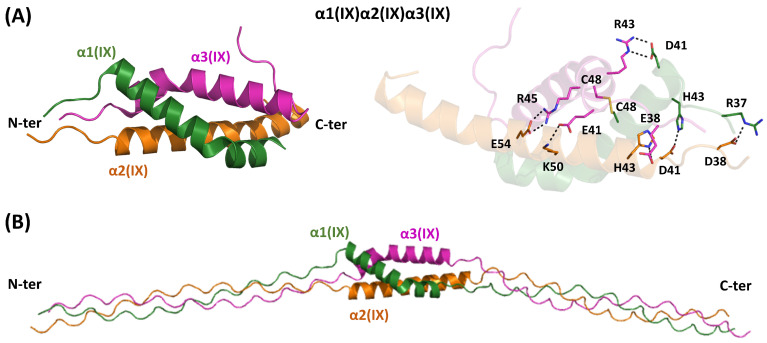
(**A**) Experimental model of the three-helix bundle formed by the NC2 domains of chains α1 (green), α2 (orange), and α3 (magenta) of collagen type IX, as extracted from the crystallographic structure (PDB ID: 5ctd). The interactions stabilizing this hetero-oligomer are shown on the right. (**B**) AF3-predicted model of the α1(IX)α2(IX)α3(IX) NC2 hetero-trimer, including triple-helix regions at both N- and C-termini. The residue ranges defining the different regions are reported in Table S1.

In these crystal structures, the NC2 domain consists of three α-helices arranged in a highly distorted triple helix bundle (Figure 1 and Figure 7A), stabilized by a disulfide bond that covalently links the α1(IX) and α3(IX) chains. The overall structure of the heterotrimer is maintained through hydrophobic interactions and a network of hydrogen bonds and electrostatic interactions (Figure 7A), which also help determine the chain specificity of the trimer. Analyzing the junction between the globular region and the triple helical structure reveals that the α2(IX) chain leads. Conversely, the α1(IX) and α3(IX) chains occupy middle and trailing positions, respectively.

To gain further insights into the role of NC2 in the triple helix assembly of type IX collagen, we performed predictions with AF3 (Figure 7B and Figure S12). We initially assessed the ability of the software to predict the structure of this non-collagenous domain. The structure of this heterotrimeric assembly strictly follows the crystallographic structure. Since the experimental structural analyses were performed by linking the N-terminal of the type IX NC2 with type I GXY sequences, which were more prone to form triple helical structures, we performed AF3 predictions using only type IX sequences. Moreover, in our tests, triple helical fragments were added to both the N- and C-terminal side of NC2 (Figure 7B). As shown in Figure S12, reliable predictions were obtained according to the pLDDT score and the PAE matrix. Using this model, we evaluated the staggering of the three chains on both sides. In the triple helix region located at the N-terminus of NC2, the order of the chains is α2(IX), α1(IX), and α3(IX), in line with the structural data reported for the mixed type I/type IX construct [77]. The inspection of the AF3 model indicates that this staggering of the chains is also observed in the triple helix located at the C-terminus of NC2.

These findings prompted us to perform predictions on the structure of the other NC domains of the protein. For the NC1 domain, a sort of triple helix bundle, which exhibits good AF3 self-assessment scores, is predicted (Figure S12). The model also highlights the proximity of some cysteine side chains, which may be involved in intermolecular disulfide bridges that can stabilize the heterotrimer. Particularly close are the side chains of the Cys662 and Cys667 of α3(IX) with subunits α1(IX)Cys901 and α2(IX)Cys669, respectively. No reliable, well-defined structures are predicted for the NC3 domain, while, as expected, a thrombospondin domain is predicted in correspondence with NC4.

## 5. Conclusions

A comprehensive understanding of the relationship between sequence, structure, and function in collagens is a highly complex scientific goal. Although the defining feature of collagen, the triple helix, is characterized by repetitive sequences and a regular structure motif, the function of these proteins depends on other, structurally distinct regions. Indeed, collagens are biomolecules whose functionality relies on the interplay of three major structural elements in proteins: intrinsically disordered, globular, and fibrous regions. This review focuses on the current state of structural characterizations of the NC regions that lack the Gly-X-Y sequence triplets but are nonetheless essential for collagen trimer assembly and supramolecular organization of all collagens exhibiting heteromeric organizations. Considering the impressive progress of predictive approaches to protein structures, the information retrieved from the survey of literature and the PDB structural database was integrated by generating three-dimensional models of NC domains that were not experimentally characterized. Collectively, the data reported here indicate that, although the precise structural basis of the chain selection and staggering is yet to be achieved in many cases, the available structural information represents a solid base for future studies aimed at unravelling this puzzling issue.

In a more general framework, the heteromeric association of chains displaying sequence and structural similarity represents an intriguing question. Studies carried out on globular proteins indicate that a sufficient level of sequence dissimilarity in the association of a polypeptide chain with the same folding is beneficial to establish interactions that favor hetero- versus homo-oligomerization [78]. Specifically, it has been found that mean sequence identity between interacting homologous subunits is only 23–24% [78]. The analysis of the overall sequence identity shown by the NC regions of the interacting chains clearly indicates that these generally retain higher similarity. This observation suggests that rather stringent sequence modifications in these domains drive the formation of heterotrimeric complexes. Moreover, the data analyzed here indicate that the increasing molecular complexity of heterotrimers transitioning from a 2:1 to a 1:1:1 stoichiometry correlates with a decrease in the sequence similarities of the involved chains. This observation suggests that selecting three different chains requires specific interactions, which are ensured by significant sequence modifications.

The combination of experimental structural data and predictive analyses offers some interesting clues about the structural features of heterotrimers formed by collagen NC regions. In fact, the available structural information shows that different structural factors drive heterotrimerization across various subgroups (Figure 1).

For fibrillar collagens (types I, V, XI, and mixed V/XI), key factors include the formation of specific disulfide bridges and electrostatic interaction patterns. The disulfide hypothesis [39] well explains the heterotrimerization of types I and V. Structural models, as described here, however, suggest that a quite different pattern of electrostatic interactions stabilizes the oligomeric interfaces of these two collagen types. In the case of type XI and the mixed type V/XI, disulfide bonds do stabilize the observed heterotrimeric associations, although other heterotrimers not yet experimentally detected are compatible with the disulfide hypothesis.

In the subgroup of collagens whose heterotrimers create supramolecular networks (types IV and VIII), available structural information provides a solid ground for the definition of the basis of the molecular and supramolecular organization. Experimental structural characterizations have provided a three-dimensional visualization of the localization of chloride ions that play a crucial role in collagen type IV assembly.

Recent AlphaFold predictions and structural analysis of type VI collagen offer strong evidence of the specific domains in the NC region of the protein that are involved in chain selection and their staggering [75]. In this context, the role of a three-helix coiled-coil motif is particularly significant, as it, with different disulfide patterns and interactions, stabilizes the three heterotrimers found in this collagen type. Insightful crystallographic studies have also revealed some fundamental elements of the chain selection process in type IX collagen [77]. As shown here, these indications could be extended to other regions of the protein using predictive approaches.

Overall, the data here further highlight the importance of combining experimental results with computational predictions that enable the study of an entire protein family [79,80,81,82], even when its members have quite different structural features. The strong structural foundation provided by this approach offers an important basis for understanding some key issues not addressed here, such as post-translational modifications and the effects of disease-causing mutations.

It should be noted, however, that the NC regions work in concert with the triple helix domain to ensure proper collagen assembly and function. Recent impressive studies have demonstrated the key role of the THD region in the chain selection and staggering process [40,41,83]. A full understanding of the interplay between NC regions and THD requires structural data on large collagen regions that, due to their intrinsic properties, were not amenable to crystallographic studies. Recent advances in cryo-EM techniques suggest that the reductionist approaches used so far could soon be overcome [75,84]. The recent application of this methodology to large collagen fragment regions allows us to foresee a fruitful development of these activities.

## Figures and Tables

**Table 1:** Overview of collagen types forming heterotrimers (I, IV, V, VI, VIII, IX, and XI). For each type, the corresponding chains, UniProtKB accession numbers, stoichiometry of homo- and hetero-assemblies, and, when available, the corresponding experimental PDB structures (release of June 2025) are reported.

Collagen Family	Type	Chains	UniProtKB	Homotrimeric Assemblies		Heterotrimeric Assemblies	
				Stoichiometry	PDB ID (Resolution, Å)	Stoichiometry	PDB ID
Fibrillar	I	α1(I)	P02452	[α1(I)]_3_	5k31 (2.20), 7e7b (2.60), 7e7d (3.20)	[α1(I)]_2_α2(I)	-
		α2(I)	P08123	-	-		
	V	α1(V)	P20908	[α1(V)]_3_	-	[α1(V)]_2_α2(V) α1(V)α2(V)α3(V)	-
		α2(V)	P05997	-	-		
		α3(V)	P25940	-	-		
	XI	α1(XI)	P12107	-	-	α1(XI)α2(XI)α3(XI)	-
		α2(XI)	P13942	-	-		
		α3(XI) ^#^	P02458	-	-		
	V/XI	-	-	-	-	[α1(XI)]_2_α2(V) α1(XI)α1(V)α3(XI)	-
Network-forming	IV	α1(IV)	P02462	[α1(IV)]_3_	5nay (1.80)	[α1(IV)]_2_α2(IV)	1li1 (1.90), 1m3d ^§^ (2.00), 1t60 ^§^ (1.50), 5nax (2.82), 6mpx (1.90)
		α2(IV)	P08572	[α2(IV)]_3_	5nb2 (2.50)		
		α3(IV)	Q01955	[α3(IV)]_3_	5nb0 (2.70)	α3(IV)α4(IV)α5(IV)	6wku (1.76)
		α4(IV)	P53420	[α4(IV)]_3_	5nb1 (2.82)		
		α5(IV)	P29400	[α5(IV)]_3_	5naz (1.85)		
		α6(IV)	Q14031	-	-	[α5(IV)]_2_α6(IV)	-
	VIII	α1(VIII)	P27658	[α1(VIII)]_3_	1o91 * (1.90)	[α1(VIII)]_2_α2(VIII) α1(VIII)[α2(VIII)]_2_	-
		α2(VIII)	P25067	[α2(VIII)]_3_			
Beaded filaments	VI	α1(VI)	P12109	-	-	α3(VI)α2(VI)α1(VI) α5(VI)α2(VI)α1(VI) α6(VI)α2(VI)α1(VI)	-
		α2(VI)	P12110	-	-		
		α3(VI)	P12111	-	-		
		α5(VI)	A8TX70	-	-		
		α6(VI)	A6NMZ7	-	-		
FACITs	IX	α1(IX)	P20849	-	-	α1(IX)α2(IX)α3(IX)	5ctd (1.60), 5cti (1.90), 5cva (2.10), 5cvb (2.25)
		α2(IX)	Q14055	-	-		
		α3(IX)	Q14050	-	-		

^#^ The α3(XI) chain shares the same sequence as the α1(II) chain but differs in its post-translational processing and cross-linking. * Source: Mus musculus; ^§^ Source: Bos taurus.

## Data Availability

No new data were created or analyzed in this study. Data sharing is not applicable to this article.

## Supplementary Materials

The following supporting information can be downloaded at: https://www.mdpi.com/article/10.3390/ijms262010134/s1.
