# Supplementary material for: Determinants of Chain Selection and Staggering in Heterotrimeric Collagens: A Comprehensive Review of the Structural Data
Source: Int J Mol Sci. 2025 Oct 18;26(20):10134. doi: 10.3390/ijms262010134 (PMC12562938; doi:10.3390/ijms262010134)
Supplement: Supplementary file 1 [file ijms-26-10134-s001.zip › ijms-3912621-supplementary.pdf]

## Supplementary Material

**Table S1.** Overview of collagen types that form heterotrimers (I, IV, V, VI, VIII, IX, and XI). For each type, the corresponding chains, sequence length, UniProtKB accession number, and residue numbering of the AF3-predicted models are reported.

| Collagen type | Chain | UniProtKB | Length | Predicted region | | Numbering of the predicted models |
|---|---|---|---|---|---|---|
| | | | | Notation | Residues | |
| I | α1(I) | P02452 | 1464 | NC1 (C-propeptide) | 1229-1464 | 11-246* |
| | α2(I) | P08123 | 1366 | | 1133-1366 | 14-247[§] |
| V | α1(V) | P20908 | 1838 | | 1609-1837 | 11-239* |
| | α2(V) | P05997 | 1499 | | 1266-1499 | 11-244* |
| | α3(V) | P25940 | 1745 | | 1514-1744 | 11-241* |
| XI | α1(XI) | P12107 | 1806 | | 1577-1805 | 11-239* |
| | α2(XI) | P13942 | 1736 | | 1541-1735 | 11-205* |
| | α3(XI)[#] | P02458 | 1487 | | 1253-1487 | 11-245* |
| IV | α1(IV) | P02462 | 1669 | NC1 | 1445-1669 | 5-229* |
| | α2(IV) | P08572 | 1712 | | 1489-1712 | 5-228* |
| | α3(IV) | Q01955 | 1670 | | 1445-1669 | 5-229* |
| | α4(IV) | P53420 | 1690 | | 1465-1690 | 5-230* |
| | α5(IV) | P29400 | 1685 | | 1461-1685 | 5-229* |
| | α6(IV) | Q14031 | 1691 | | 1467-1691 | 5-229* |
| VIII | α1(VIII) | P27658 | 744 | NC1 | 614-744 | 1-131 |
| | α2(VIII) | P25067 | 703 | | 573-703 | 1-131 |
| VI | α1(VI) | P12109 | 1028 | THD (578-592)-C1+C2 (593-1028) | 578-1028 | 1-451 |
| | α2(VI) | P12110 | 1019 | THD (576-590)-C1+C2 (591-1019) | 576-1019 | 1-444 |
| | α3(VI) | P12111 | 3177 | THD (2361-2375)-C1+C2 (2376-2820) | 2361-2820 | 1-460 |
| | α5(VI) | A8TX70 | 2615 | THD (1719-1728)-C1+C2 (1729-2159) | 1719-2159 | 1-441 |
| | α6(VI) | A6NMZ7 | 2263 | THD (1716-1725)-C1+C2 (1726-2263) | 1716-2171 | 1-456 |
| IX | α1(IX) | P20849 | 921 | NC1 | 899-921 | 1-23 |
| | | | | NC2 | 757-784 | 39-66 |
| | | | | THD+NC2+THD | 727-816 | 9-98 |
| | α2(IX) | Q14055 | 689 | NC1 | 662-689 | 1-28 |
| | | | | NC2 | 520-547 | 39-66 |
| | | | | THD-NC2-THD | 490-579 | 9-98 |
| | α3(IX) | Q14050 | 684 | NC1 | 660-684 | 1-25 |
| | | | | NC2 | 520-547 | 39-66 |
| | | | | THD+NC2+THD | 490-580 | 9-99 |

*for these AF3-predicted models, the numbering follows that reported in the corresponding PDB entry or in the PDB of homologous structures. Specifically, the numbering follows the entries 5k31 (types I, V, and XI), 1li1 (type IV), and 5ctd (type IX).

[§]the numbering follows that reported in the molecular modelling study by Sharma et al. [27]

[#]The α3(XI) chain shares the same sequence as the α1(II) chain but differs in its post-translational processing and cross-linking.

**Table S2.** Pairwise sequence identity between the NC1 domains (see Table S1 for the residue ranges) of heterotrimeric fibrillar collagens (types I, V, and XI). Values above the diagonal (right side) indicate the percentage of sequence identity, whereas values below the diagonal (left side) report the number of aligned residues used for the calculation.

| | **α1(I)** | **α2(I)** | **α1(V)** | **α2(V)** | **α3(V)** | **α1(XI)** | **α2(XI)** | **α3(XI)** |
|---|---|---|---|---|---|---|---|---|
| **α1(I)** | | 63.2 | 43.6 | 61.1 | 38.7 | 44.9 | 38.1 | 71.9 |
| **α2(I)** | 234 | | 39.6 | 57.8 | 37.0 | 42.4 | 33.9 | 67.8 |
| **α1(V)** | 236 | 235 | | 40.2 | 53.9 | 78.2 | 51.5 | 44.3 |
| **α2(V)** | 234 | 232 | 234 | | 35.6 | 39.3 | 36.1 | 63.1 |
| **α3(V)** | 235 | 235 | 230 | 233 | | 53.5 | 43.5 | 40.4 |
| **α1(XI)** | 236 | 236 | 229 | 234 | 230 | | 43.2 | 45.8 |
| **α2(XI)** | 236 | 233 | 229 | 233 | 230 | 229 | | 38.3 |
| **α3(XI)** | 235 | 233 | 235 | 233 | 235 | 236 | 235 | |

**Table S3.** Pairwise sequence identity between the NC1 domains (see Table S1 for the residue ranges) of collagen type IV chains. Values above the diagonal (right side) indicate the percentage of sequence identity, whereas values below the diagonal (left side) report the number of aligned residues used for the calculation.

| | **α1(IV)** | **α2(IV)** | **α3(IV)** | **α4(IV)** | **α5(IV)** | **α6(IV)** |
|---|---|---|---|---|---|---|
| **α1(IV)** | | 63.8 | 71.9 | 59.6 | 83.6 | 62.7 |
| **α2(IV)** | 224 | | 60.4 | 71.4 | 63.4 | 76.9 |
| **α3(IV)** | 224 | 225 | | 54.0 | 71.0 | 56.9 |
| **α4(IV)** | 228 | 224 | 226 | | 59.2 | 68.8 |
| **α5(IV)** | 225 | 224 | 224 | 228 | | 64.0 |
| **α6(IV)** | 225 | 225 | 225 | 224 | 225 | |

**Table S4.** Pairwise sequence identity between the NC2 domains (see Table S1 for the residue ranges) of collagen type IX chains. Values above the diagonal (right side) indicate the percentage of sequence identity, whereas values below the diagonal (left side) report the number of aligned residues used for the calculation.

| | **α1(IX)** | **α2(IX)** | **α3(IX)** |
|---|---|---|---|
| **α1(IX)** | | 53.8 | 37.0 |
| **α2(IX)** | 26 | | 29.6 |
| **α3(IX)** | 27 | 27 | |

**Table S5.** Pairwise sequence identity between the first two C-terminal vWF-A-like subdomains (C1–C2) of collagen type VI chains (see Table S1 for the residue ranges). Values above the diagonal (right side) indicate the percentage of sequence identity, whereas values below the diagonal (left side) report the number of aligned residues used for the calculation. No acceptable alignment can be obtained for α6(VI) with α2(VI) and α5(VI).

| | **α1(VI)** | **α2(VI)** | **α3(VI)** | **α5(VI)** | **α6(VI)** |
|---|---|---|---|---|---|
| **α1(VI)** | | 31.7 | 20.1 | 16.3 | 21.4 |
| **α2(VI)** | 420 | | 24.1 | 19.8 | - |
| **α3(VI)** | 422 | 320 | | 32.9 | 32.7 |
| **α5(VI)** | 410 | 440 | 414 | | - |
| **α6(VI)** | 415 | - | 416 | - | |

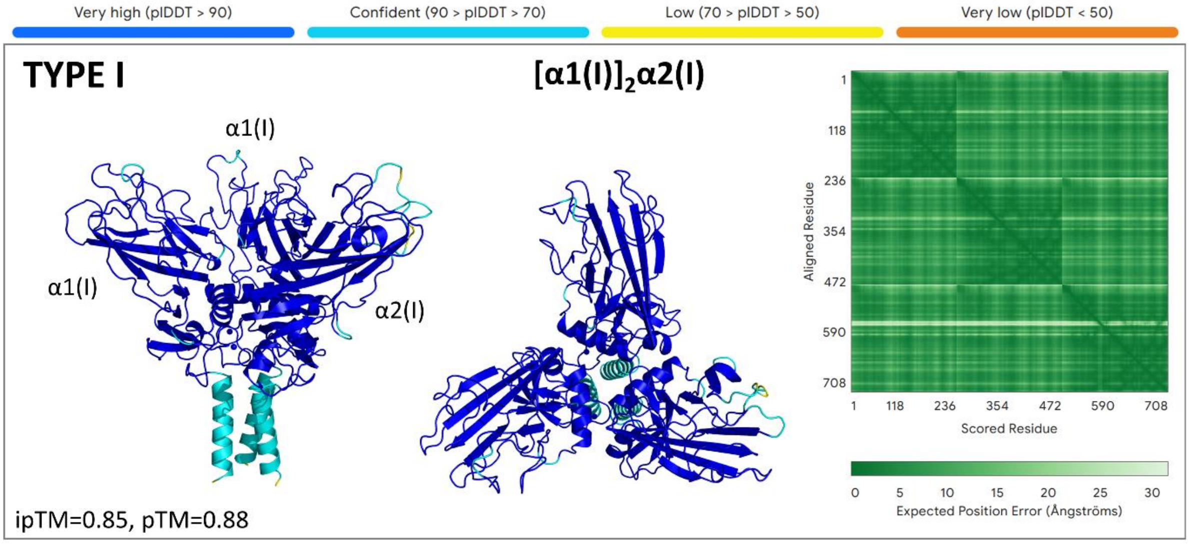


**Figure S1.** Cartoon representation and PAE matrices of the AF3-predicted heterotrimer of the NC1 domains of collagen type I. Structural models are colored following the AF3 per-residue confidence metric (pLDDT). The ipTM and pTM scores are also reported for each model. The residue ranges defining the different regions are reported in Table S1.

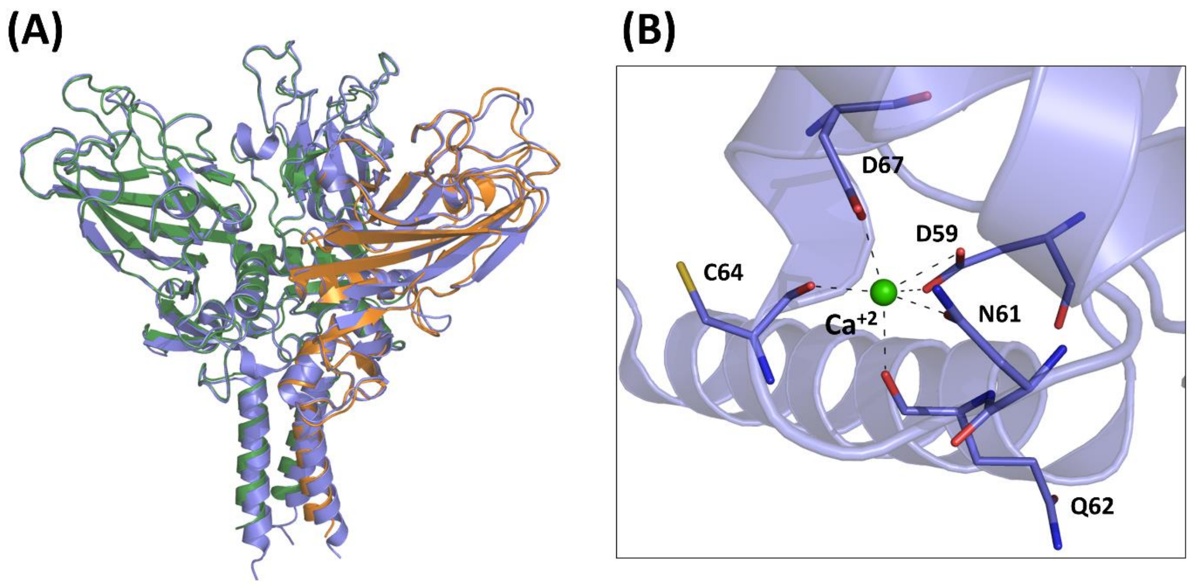


**Figure S2.** Superimposition of the AF3-predicted model of the $[\alpha1(I)]_2\alpha2(I)$ heterotrimer ($\alpha1(I)$ in green and $\alpha2(I)$ in orange) with the crystallographic structure of the $[\alpha1(I)]_3$ homotrimer shown in violet (PDB ID: 5k31) (A). Calcium ion coordination in the crystallographic model of $[\alpha1(I)]_3$ (B).

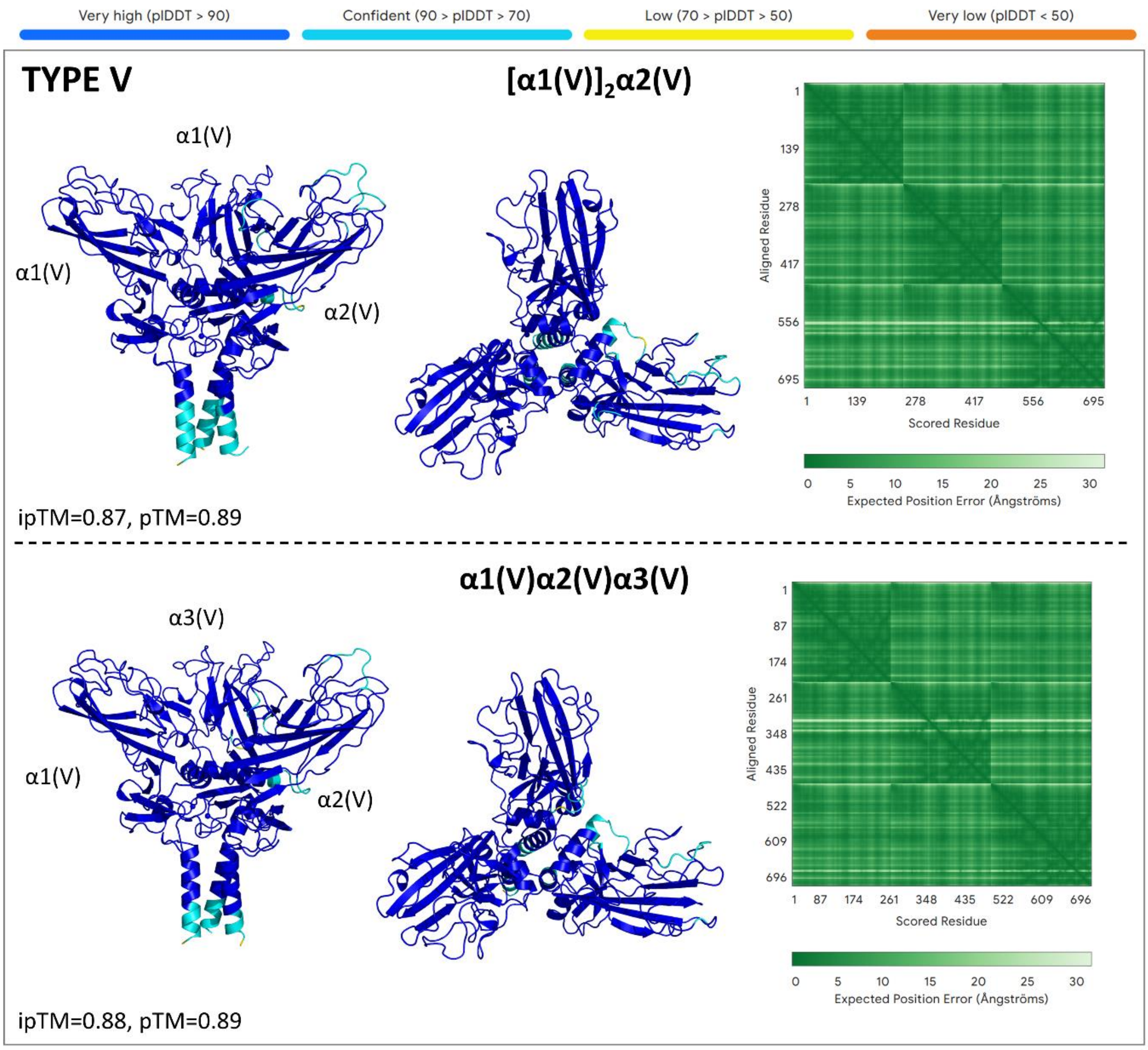


**Figure S3.** Cartoon representation and PAE matrices of AF3-predicted heterotrimers of the NC1 domains of collagen type V. Structural models are colored following the AF3 per-residue confidence metric (pLDDT). The ipTM and pTM scores are also reported for each model. The residue ranges defining the different regions are reported in Table S1.

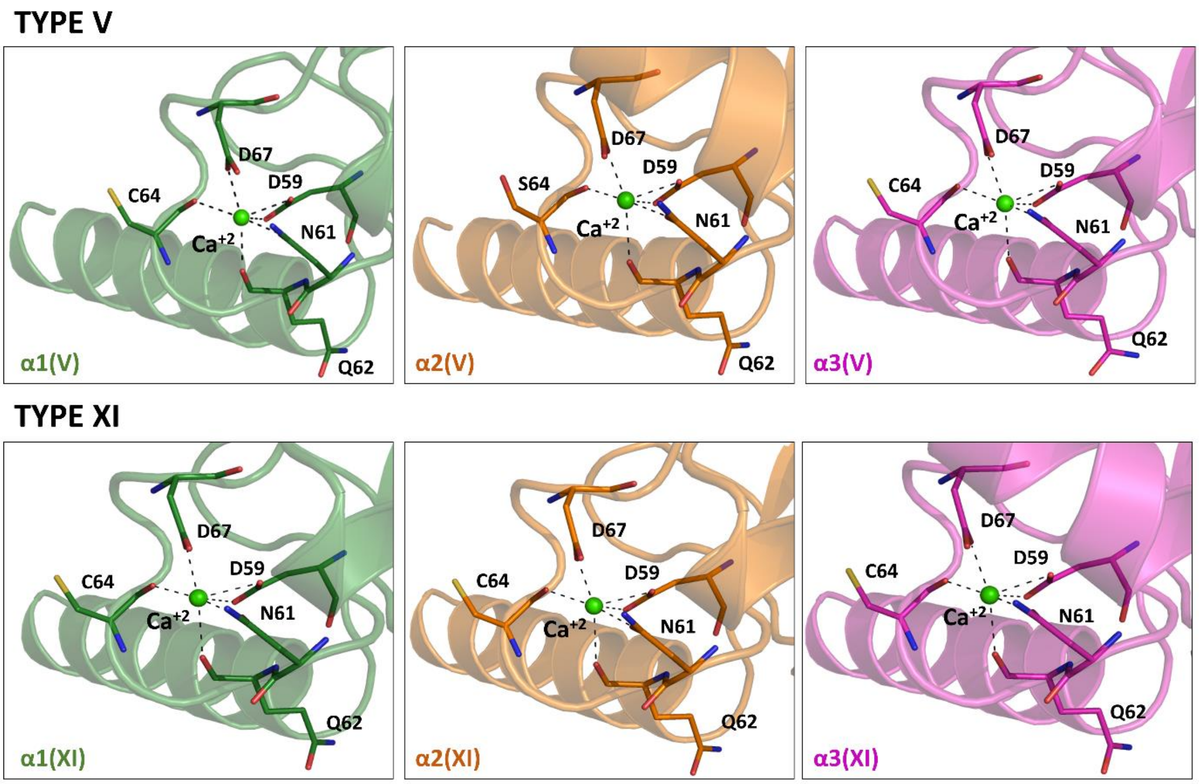


**Figure S4.** Calcium ion coordination in chains α1, α2, and α3 in the AF3-predicted models of the heterotrimers formed by the NC1 domains of collagen types V and XI.

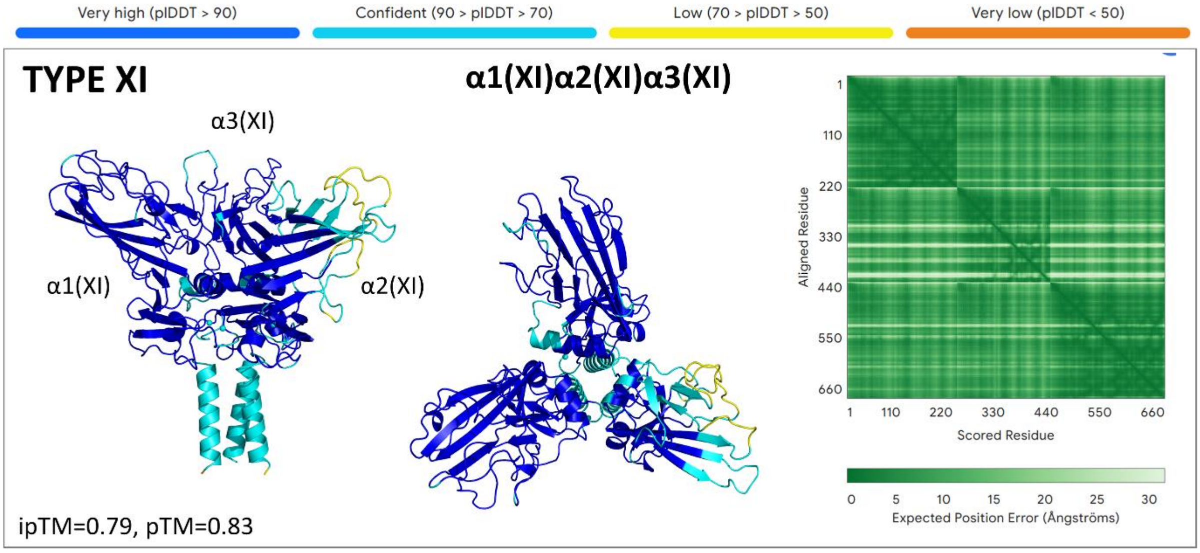


**Figure S5.** Cartoon representation and PAE matrices of the AF3-predicted heterotrimer of the NC1 domains of collagen type XI. Structural models are colored following the AF3 per-residue confidence metric (pLDDT). The ipTM and pTM scores are also reported for each model. The residue ranges defining the different regions are reported in Table S1.

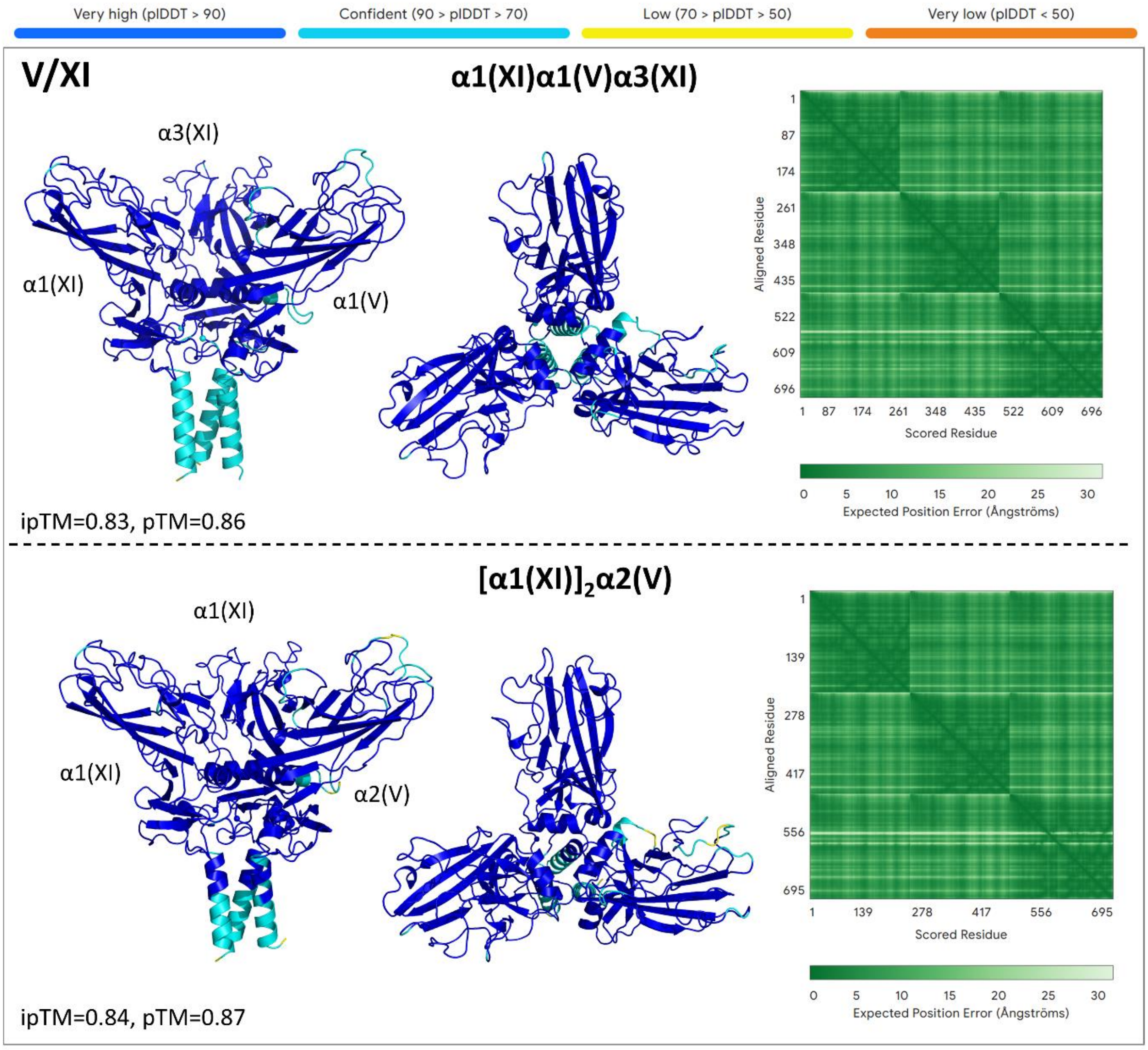


**Figure S6.** Cartoon representation and PAE matrices of AF3-predicted mixed heterotrimers of the NC1 domains of collagen types V and XI. Structural models are colored following the AF3 per-residue confidence metric (pLDDT). The ipTM and pTM scores are also reported for each model. The residue ranges defining the different regions are reported in Table S1.

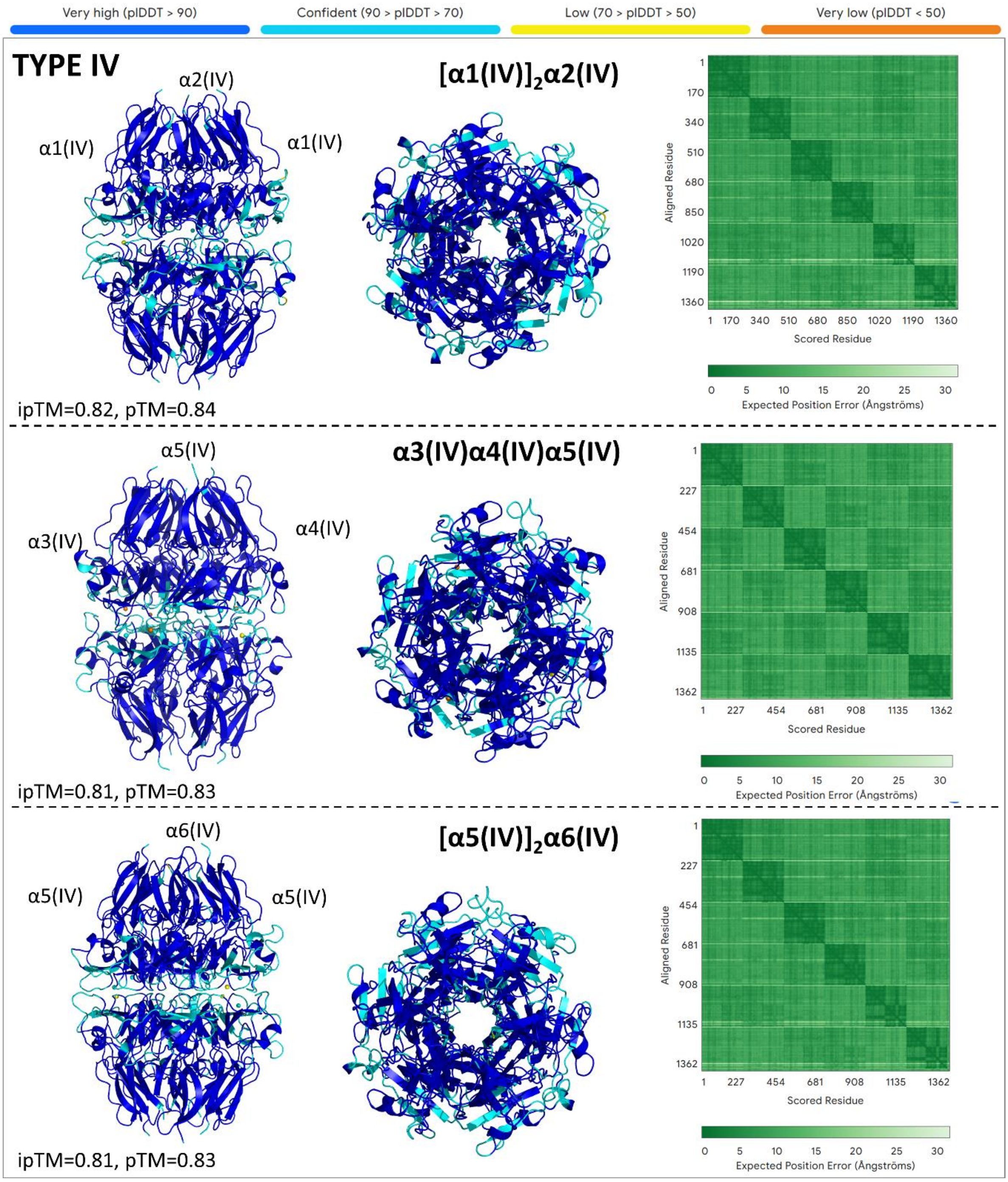


**Figure S7.** Cartoon representation and PAE matrices of AF3-predicted hetero-oligomers of the NC1 domains of collagen type IV. Structural models are colored following the AF per-residue confidence metric (pLDDT). The ipTM and pTM scores are also reported for each model. The residue ranges defining the different regions are reported in Table S1.

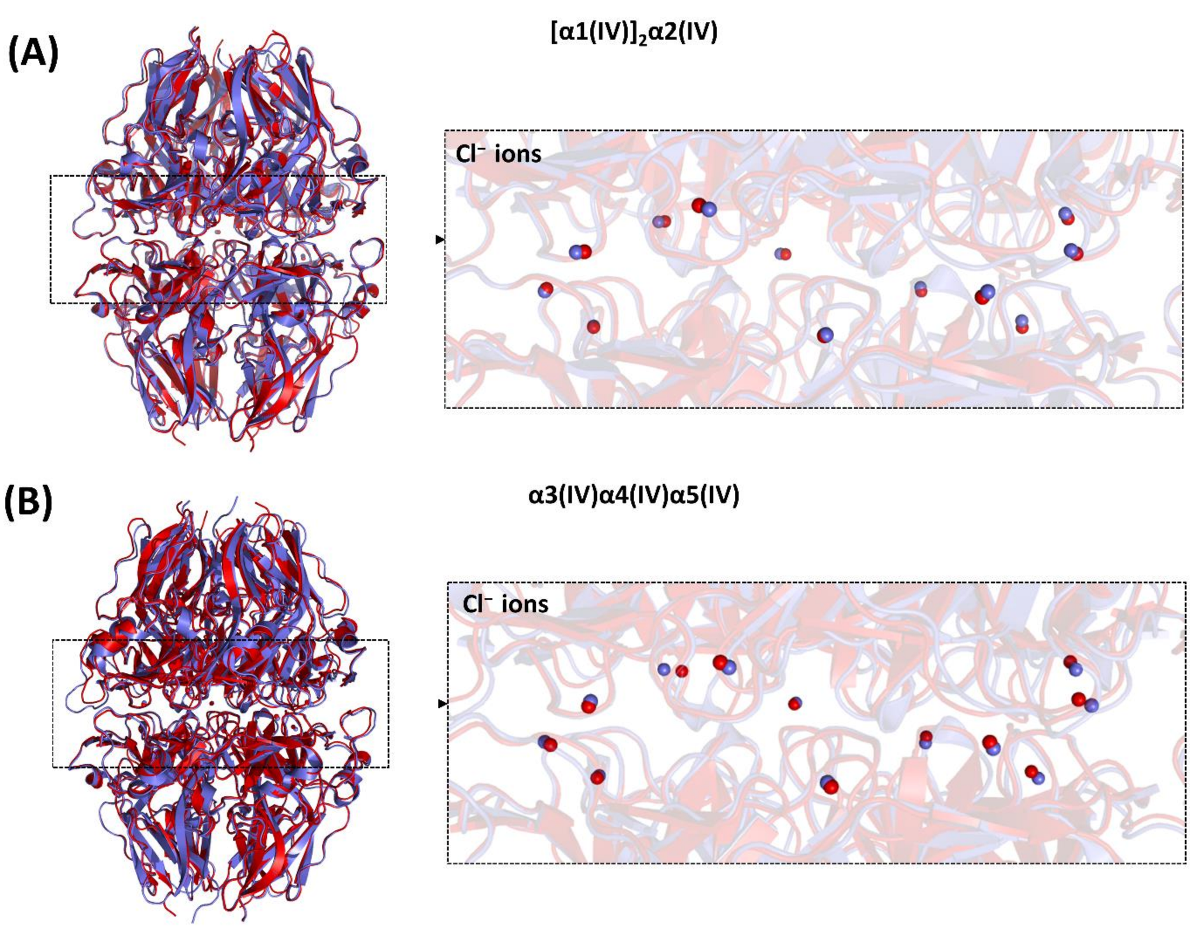


**Figure S8.** Superimposition of the AF3-predicted models of the [α1(IV)]$_2$α2(IV) (A) and α3(IV)α4(IV)α5(IV) (B) hetero-oligomers (in red) formed by the NC1 domains of collagen type IV, with their respective crystallographic structures shown in violet, PDB IDs: 6mpx (A) and 6wku (B). The right panels show a zoomed view of the interfaces between the two trimers within the hexamers, highlighting chloride ions depicted as spheres.

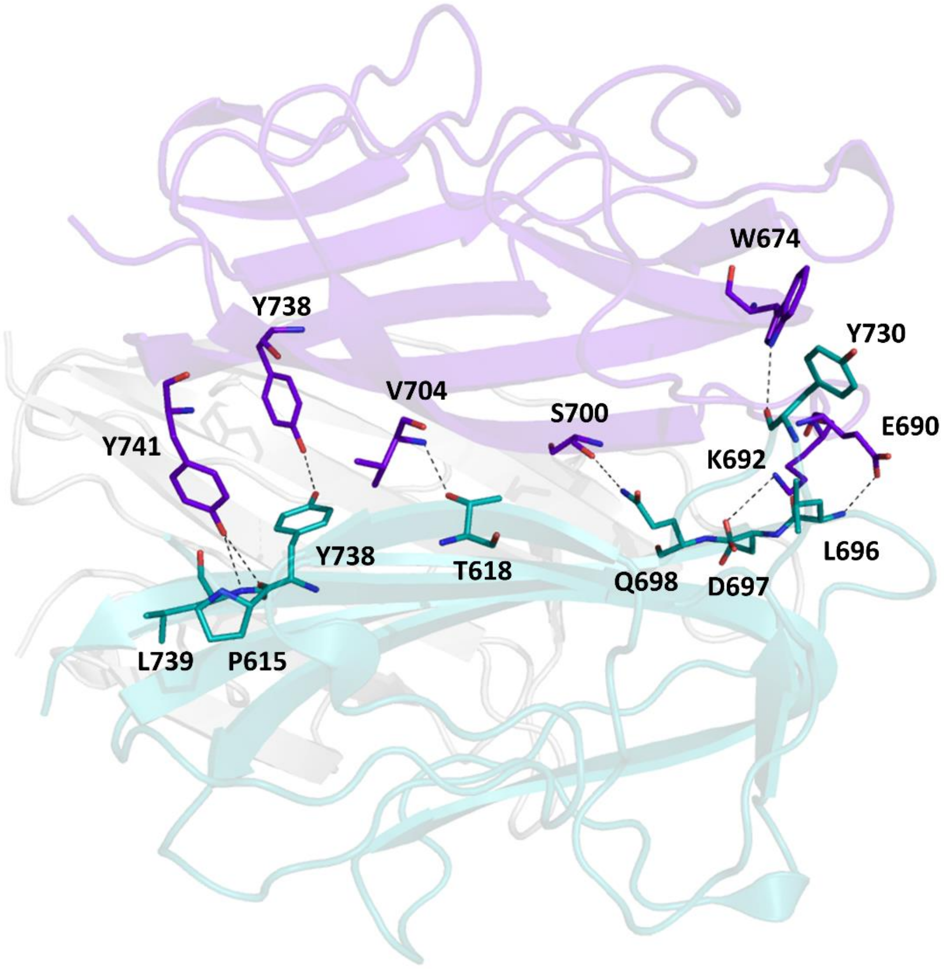


**Figure S9.** Stabilizing interactions at the homo-interfaces of the NC1 domains in the crystallographic structure of α1(VIII)]$_3$ (PDB ID: 1o91). The interface between chains A (cyan) and B (violet) is shown.

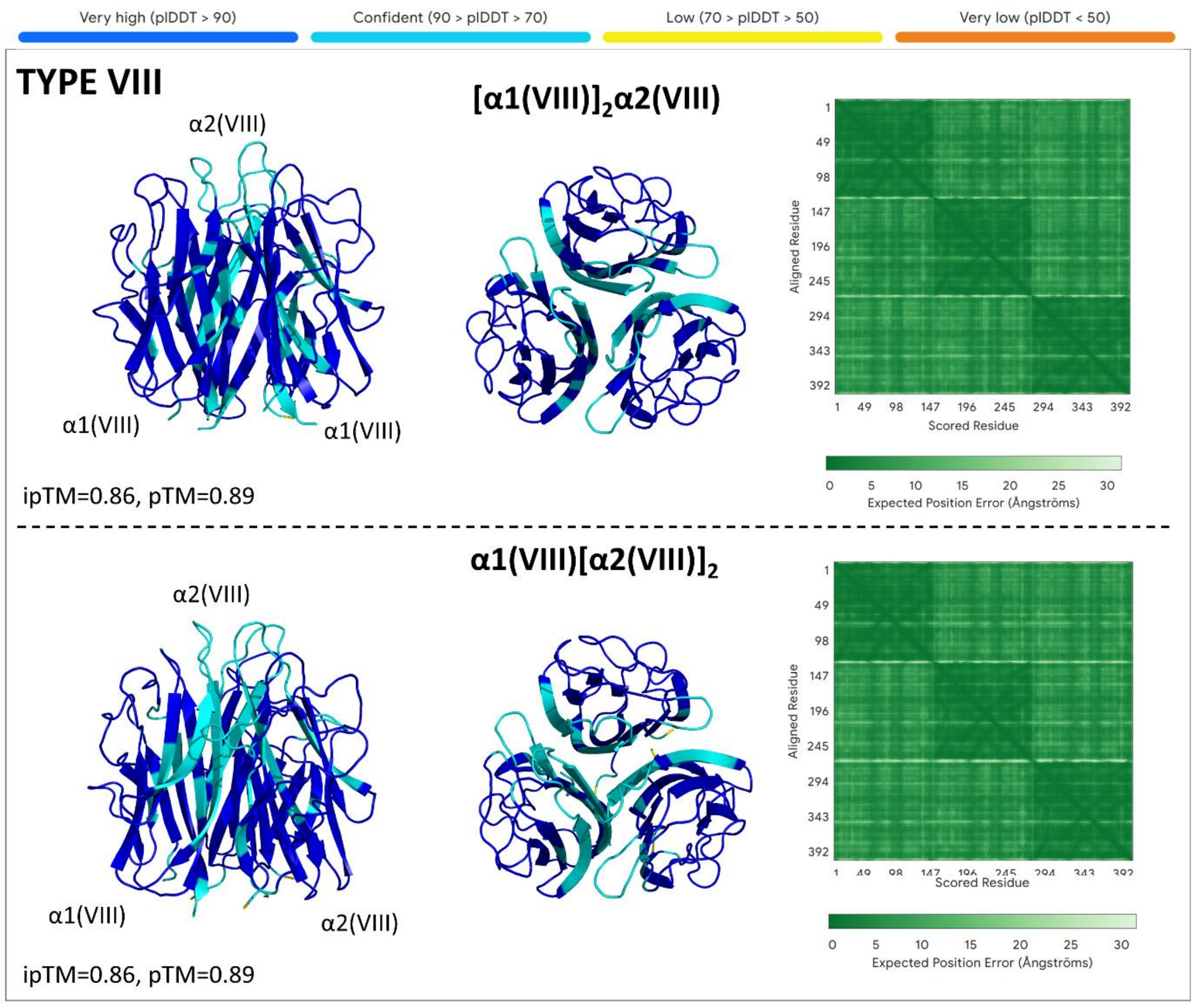


**Figure S10.** Cartoon representation and PAE matrices of AF3-predicted hetero-oligomers of the NC1 domains of collagen type VIII. Structural models are colored following the AF per-residue confidence metric (pLDDT). The ipTM and pTM scores are also reported for each model. The residue ranges defining the different regions are reported in Table S1.

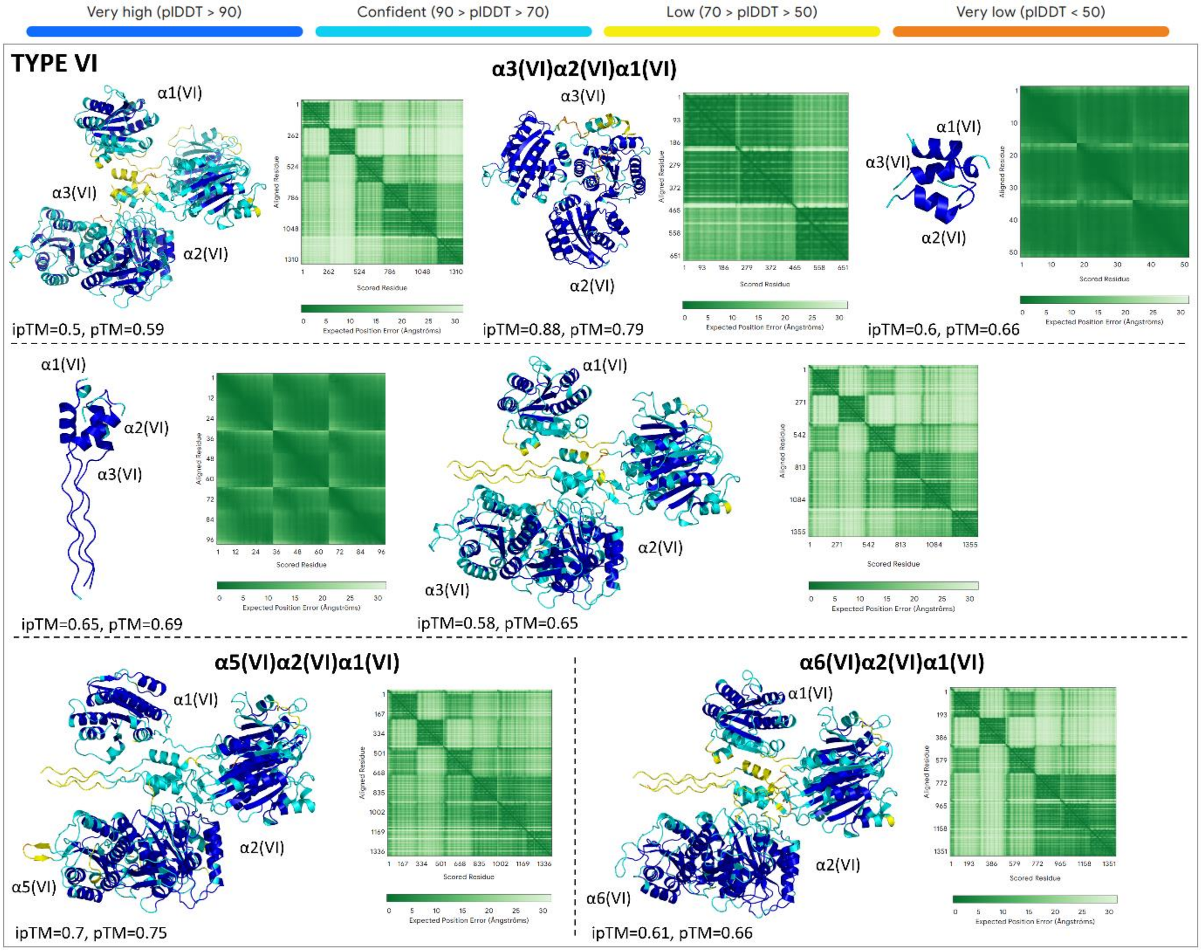


**Figure S11.** Cartoon representation and PAE matrices of AF3-predicted hetero-oligomers formed by the C1+C2 domains of collagen type VI. Structural models are colored following the AF per-residue confidence metric (pLDDT). The ipTM and pTM scores are also reported for each model. The residue ranges defining the different regions are reported in Table S1.

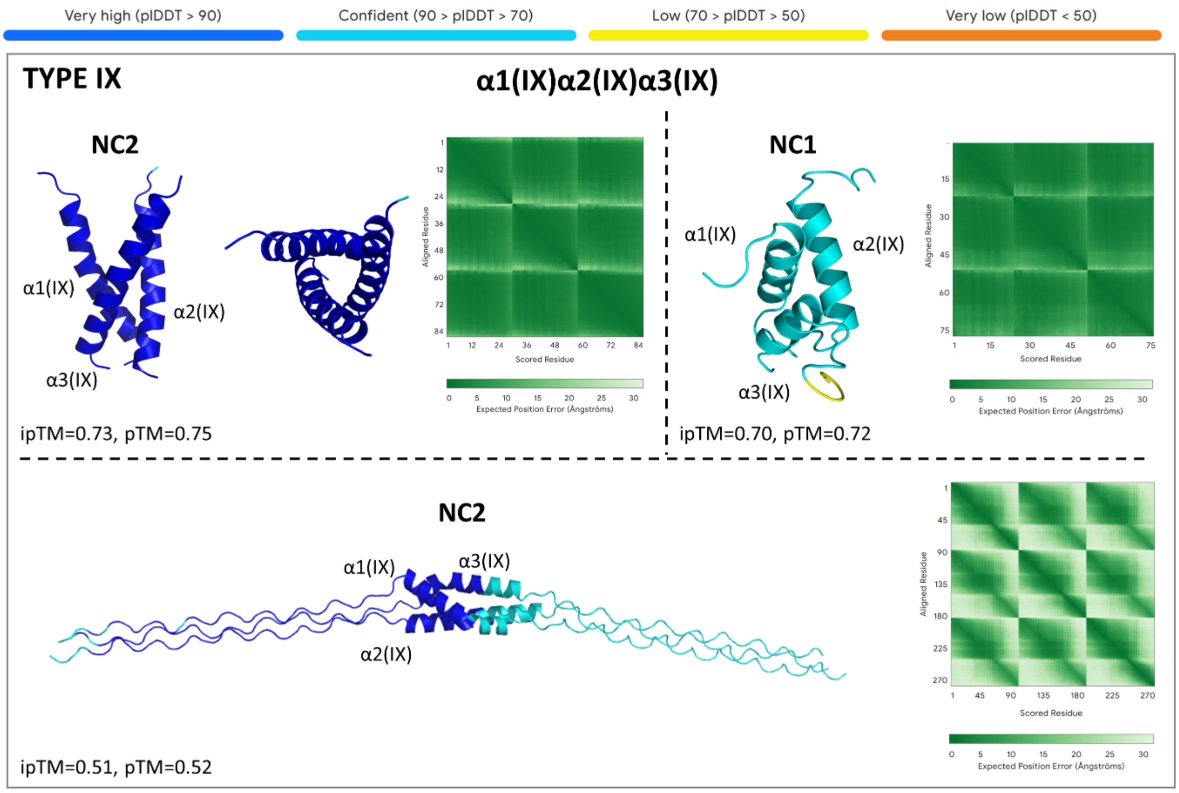


**Figure S12.** Cartoon representation and PAE matrices of AF3-predicted hetero-trimers formed by either the NC1 or the NC2 domains of collagen type IX. Structural models are colored according to the AF per-residue confidence metric (pLDDT). The ipTM and pTM scores are also reported for each model. The residue ranges defining the different regions are reported in Table S1.
